# When Pathways Converge: Iron, Lipid Peroxidation, and α‐Synuclein in Ferroptosis‐Driven Dopaminergic Neurodegeneration

**DOI:** 10.1111/jnc.70438

**Published:** 2026-04-13

**Authors:** Carmem L. Sperlich, Brent R. Stockwell, Marcelo Farina

**Affiliations:** ^1^ Department of Biochemistry, Center of Biological Sciences Federal University of Santa Catarina Florianópolis Santa Catarina Brazil; ^2^ Graduate Program on Neurosciences, Center of Biological Sciences Federal University of Santa Catarina Florianópolis Santa Catarina Brazil; ^3^ Department of Biological Sciences Columbia University New York New York USA; ^4^ Department of Chemistry Irving Institute for Cancer Dynamics, and Data Science Institute New York New York USA; ^5^ Department of Pathology and Cell Biology, Herbert Irving Comprehensive Cancer Center Columbia University Digestive and Liver Disease Research Center, Vagelos College of Physicians and Surgeons, Columbia University Irving Medical Center New York New York USA; ^6^ Graduate Program on Biochemistry, Center of Biological Sciences Federal University of Santa Catarina Florianópolis Santa Catarina Brazil

**Keywords:** dopaminergic neurons, ferroptosis, iron, neurodegeneration, Parkinson's disease

## Abstract

The selective degeneration of dopaminergic neurons is a hallmark of Parkinson's disease and related disorders. While multiple cell death pathways have been implicated, ferroptosis has recently emerged as a critical mechanism. This iron‐dependent form of regulated cell death is driven by the accumulation of phospholipid hydroperoxides, leading to oxidative membrane damage. Dopaminergic neurons are intrinsically vulnerable to ferroptosis due to their high iron content, active dopamine metabolism (a source of reactive oxygen species), and relatively low antioxidant defenses. Here we synthesize evidence linking ferroptosis to dopaminergic neurodegeneration in Parkinson's disease and related conditions, detailing the molecular mechanisms involving iron dyshomeostasis, lipid peroxidation, and α‐synuclein pathology. We further evaluate growing preclinical data demonstrating that pharmacological inhibition of ferroptosis is neuroprotective and discuss the clinical implications, therapeutic potential, and ongoing challenges of translating these findings into effective treatments for patients.

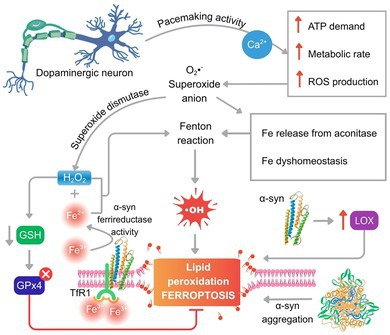

## Graphical abstract

Dopaminergic neurons are particularly susceptible to ferroptosis. Pacemaking activity–driven calcium (Ca^2+^) influx increases metabolic demand and reactive oxygen species (ROS) production, promoting iron release from aconitase. Alpha‐synuclein (α‐syn) enhances iron uptake via transferrin receptor 1 (TfR1) and exhibits ferrireductase activity, converting ferric iron (Fe^3+^) to ferrous iron (Fe^2+^), thereby fueling the Fenton reaction and generation of the hydroxyl radical (˙OH), a key molecule initiating lipid peroxidation. Alpha‐synuclein also upregulates lipoxygenase 15 (LOX), supporting lipid peroxidation and ferroptosis. Hydrogen peroxide (H_2_O_2_) accumulation can deplete glutathione (GSH), limiting the substrate availability for glutathione peroxidase 4 (GPX4) and hindering its catalytic efficiency, leading to lipid peroxidation and ultimately ferroptotic neuronal death.

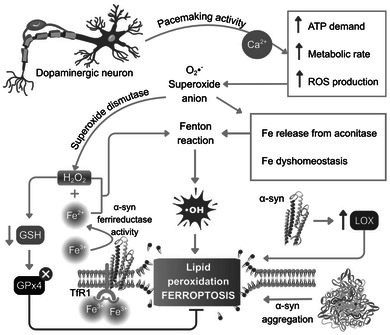

**Abbreviations**
4‐HNE: 4‐hydroxy‐2‐nonenal
6‐OHDA: 6‐hydroxydopamine
AA: arachidonic acid
AADC: aromatic L‐amino acid decarboxylase
AdA: adrenic acid
ADHD: attention deficit hyperactivity disorder
ALOX: arachidonate lipoxygenase
ARE: antioxidant response element
BBB: blood–brain barrier
BCEC: brain capillary endothelial cell
BH_2_
: dihydrobiopterin
BH_4_
: tetrahydrobiopterin
CoA: coenzyme A
CoQ_10_
: coenzyme Q_10_
CoQ_10_H_2_
: ubiquinol
CSF: cerebrospinal fluid
DAT: dopamine transporter
DHFR: dihydrofolate reductase
DMT1: divalent metal transporter 1
DOPAL: 3,4‐dihydroxyphenylacetaldehyde
DPP‐4: dipeptidyl peptidase‐4
EGCG: epigallocatechin‐3‐gallate
ER: endoplasmic reticulum
Fe^2+^
: ferrous iron
Fe^3+^
: ferric iron
Fer‐1: ferrostatin‐1
FPN1: ferroportin
FSP1: ferroptosis suppressor protein 1
FTH1: ferritin heavy chain
FTL: ferritin light chain
FTMT: mitochondrial ferritin
GCH1: GTP cyclohydrolase 1
GGC: gamma‐glutamylcysteine
GPx: glutathione peroxidase
GPx4: glutathione peroxidase 4
GSH: glutathione
GSSG: oxidized glutathione
H_2_O_2_
: hydrogen peroxide
HVA: homovanillic acid
iPSC: induced pluripotent stem cell
IRE: iron‐responsive element
IRP: iron regulatory protein
KEAP1: Kelch‐like ECH‐associated protein 1
L‐DOPA: levodopa
LIP: labile iron pool
LO˙: lipid alkoxyl radical
LOO˙: lipid peroxyl radical
LOOH: lipid hydroperoxide
LOX: lipoxygenase
LPS: lipopolysaccharide
MDA: malondialdehyde
MPAN: mitochondrial membrane protein‐associated neurodegeneration
MPP+: 1‐methyl‐4‐phenylpyridinium
MPTP: 1‐methyl‐4‐phenyl‐1,2,3,6‐tetrahydropyridine
MUFA: monounsaturated fatty acid
NAC: N‐acetylcysteine
NBIA: neurodegeneration with brain iron accumulation
NCOA4: nuclear receptor coactivator 4
NRF2: nuclear factor erythroid 2‐related factor 2
NTBI: non‐transferrin‐bound iron
PD: Parkinson's disease
PE: phosphatidylethanolamine
PEBP1: phosphatidylethanolamine‐binding protein 1
PKAN: pantothenate kinase‐associated neurodegeneration
PL: phospholipid
PLOOH: phospholipid hydroperoxide
POR: cytochrome P450 oxidoreductase
PUFA: polyunsaturated fatty acid
ROS: reactive oxygen species
SNpc: substantia nigra pars compacta
SOD: superoxide dismutase
Tf: transferrin
Tf‐Fe: transferrin‐bound iron
TfR1: transferrin receptor 1
TfR2: transferrin receptor 2
TH: tyrosine hydroxylase
TLR: Toll‐like receptor
VMAT2: vesicular monoamine transporter 2
VTA: ventral tegmental area
xCT: cystine/glutamate antiporter light chain subunit

## 1 Introduction

Dopamine is a critical monoamine neurotransmitter that orchestrates a vast array of essential processes. By signaling through a family of G‐protein‐coupled receptors, it exerts modulatory control over functions ranging from motor coordination and hormone secretion to motivation, reward, arousal, and higher‐order executive functions (Beaulieu and Gainetdinov 2011). This broad neuromodulatory influence is executed through several distinct dopaminergic pathways, the major ones being the nigrostriatal, mesolimbic, mesocortical, and tuberoinfundibular pathways (Ben‐Jonathan 2020).

The integrity of these dopaminergic pathways is fundamental to neurological health, and their dysregulation is a hallmark of numerous neuropsychiatric and neurodegenerative diseases (Klein et al. 2019). In particular, the selective and progressive degeneration of the nigrostriatal pathway represents a fundamental pathological hallmark of distinct neurodegenerative disorders, such as Parkinson's disease (PD) (Cramb et al. 2023), as well as other related conditions, including dementia with Lewy bodies (Durcan et al. 2023) and neuroferritinopathy (Vidal et al. 2004). The selective vulnerability of SNpc dopaminergic neurons is not random but is attributed to a unique set of biochemical and physiological characteristics (Mamelak 2018; Mattson 2007; Meiser et al. 2013), which converge to trigger different modes of regulated cell death, including classical apoptosis (Burguillos et al. 2011) and necroptosis (Hu et al. 2019).

More recently, ferroptosis has emerged as a significant and distinct mechanism driving dopaminergic cell loss (Awasthi et al. 2025). This iron‐dependent form of regulated cell death is characterized by the pervasive accumulation of phospholipid hydroperoxides, leading to oxidative damage of cellular membranes and subsequent lytic cell death (Dixon et al. 2012; Stockwell 2022). Dopaminergic neurons of the SNpc exhibit a confluence of intrinsic properties that create a uniquely permissive environment for ferroptosis, rendering the mechanistic link between this cell death pathway and their selective vulnerability especially compelling. Although this unique physiology confers intrinsic risk factors that increase susceptibility to ferroptosis, it also highlights the inhibition of this pathway as a potentially promising neuroprotective strategy.

The convergence of these risk factors, which collectively establish a pronounced susceptibility to ferroptosis, justifies why experimental evidence from cellular and animal models consistently demonstrates that pharmacological inhibition of ferroptosis confers significant neuroprotection against dopaminergic degeneration (Li, Wang, et al. 2023; Sun et al. 2025; Xu et al. 2025; Zhao et al. 2024). Furthermore, clinical and postmortem studies conducted before the coinage of the term *ferroptosis* identified, predominantly in dopaminergic‐neuron‐enriched structures of the PD brain such as the SNpc, molecular changes such as iron dyshomeostasis and lipid peroxidation that are now recognized as hallmarks of this cell death pathway (Dexter, Carter, et al. 1989; Pearce et al. 1997). Here, we bring updated knowledge concerning the intricate relationship between ferroptosis and dopaminergic neurodegeneration. We provide a detailed examination of the molecular mechanisms underpinning this connection, evaluate the clinical and pathological evidence from human studies, and critically discuss the promise of emerging therapeutic strategies that target the ferroptotic pathway.

## 2 Overview of Dopaminergic Neurotransmission and the Unique Vulnerability of Dopaminergic Neurons

### 2.1 Integrated Overview of Dopaminergic Neurotransmission, Pathways, and Associated Disorders

Dopamine is a catecholamine neurotransmitter essential for motor control, reward, cognition, and endocrine regulation (Volkow and Morales 2015). Synthesized in neurons from tyrosine via tyrosine hydroxylase (TH; the rate‐limiting step) and aromatic L‐amino acid decarboxylase (AADC), dopamine is stored in vesicles by the vesicular monoamine transporter 2 (VMAT2) (Daubner et al. 2011; Hornykiewicz 2017). Synaptic release allows dopamine to activate postsynaptic receptors or presynaptic autoreceptors. Signal termination occurs primarily via reuptake by the dopamine transporter (DAT) (Channer et al. 2023; Segura‐Aguilar et al. 2014). Intracellular degradation proceeds through convergent MAO/COMT pathways, culminating in homovanillic acid (HVA), a marker of dopamine turnover (Eisenhofer et al. 2004).

Dopamine signals through five receptor subtypes grouped into two families. The D1‐like family (D1, D5) couples to Gs proteins, stimulating cAMP production. The D2‐like family (D2, D3, D4) couples to Gi/o proteins, inhibiting adenylyl cyclase and modulating ion channels (Beaulieu and Gainetdinov 2011; Sidhu and Niznik 2000). Receptor distribution is widespread but distinct: D1 receptors are abundant in the cortex and striatum, supporting cognitive and motor functions, while D2 receptors are prominent in striatal, hypothalamic, and pituitary regions, influencing motor and neuroendocrine control (De Keyser et al. 1988).

Dopaminergic function is mediated by discrete pathways originating mainly in the substantia nigra pars compacta (SNpc) and ventral tegmental area (VTA):

- Nigrostriatal Pathway (SNpc to dorsal striatum): Critical for motor control and coordination. Its degeneration is the core pathological feature of Parkinson's disease (PD) (Dexter et al. 1986; Jamwal and Kumar 2019).
- Mesolimbic Pathway (VTA to nucleus accumbens, amygdala, hippocampus): Central to reward processing, motivation, and addiction. Hyperactivity is implicated in positive symptoms of schizophrenia (Volkow and Morales 2015).
- Mesocortical Pathway (VTA to prefrontal cortex): Modulates executive functions such as working memory and attention; its hypoactivity contributes to cognitive deficits in schizophrenia and ADHD (Chandler et al. 2014; Del Campo et al. 2011).
- Tuberoinfundibular Pathway (Hypothalamus to pituitary): Inhibits prolactin secretion; its disruption causes hyperprolactinemia (Rucker and Ikuta 2019).

Dysfunction in these circuits underlies numerous disorders. In schizophrenia, mesolimbic hyperactivity and mesocortical hypoactivity correlate with positive and negative symptoms, respectively (Birtwistle and Baldwin 1998). Addiction involves drug‐induced hyperdopaminergia in the mesolimbic pathway, reinforcing compulsive behavior (Wise and Jordan 2021). ADHD is linked to impaired dopaminergic signaling in prefrontal circuits (Del Campo et al. 2011).

Movement disorders, particularly parkinsonian syndromes, are strongly tied to dopaminergic disruption. PD, the most common cause, is characterized by progressive SNpc neuron loss and nigrostriatal pathway degeneration, leading to striatal dopamine depletion and motor symptoms (bradykinesia, rigidity, tremor, postural instability) (Postuma et al. 2015). Motor signs appear only after ~70% of nigral neurons are lost (Postuma and Berg 2016). PD prevalence increases with age, affecting ~1% of those over 60, with global cases projected to rise sharply (Ou et al. 2021; Su et al. 2025).

The neuropathological hallmark of PD is intraneuronal aggregates of α‐synuclein, forming Lewy bodies (Przedborski 2017). While α‐synuclein's native function involves synaptic vesicle regulation and neurotransmitter release, its aggregation drives neuronal dysfunction and death (Bendor et al. 2013; Emamzadeh 2016). α‐Synuclein pathology in non‐motor regions explains prodromal symptoms (e.g., sleep disorders, autonomic dysfunction) that can precede motor onset (Chaudhuri et al. 2006). Other conditions such as dementia with Lewy bodies, multiple system atrophy, and drug‐induced parkinsonism also disrupt dopaminergic signaling and present with parkinsonism (Aarsland et al. 2021). Furthermore, disorders such as restless legs syndrome and neurodegeneration with brain iron accumulation (NBIA) share mechanistic links to dopaminergic dysfunction (Levi et al. 2024; Michaud et al. 2002).

In summary, dopamine neurotransmission integrates motor, cognitive, emotional, and endocrine functions through its precise synthesis, receptor signaling, and distinct anatomical pathways. Its disruption is central to a spectrum of disorders, with PD representing a prime example of nigrostriatal pathway failure. Critically, the synthesis and metabolism of dopamine within SNpc neurons do not merely support neurotransmission; they establish a biochemical environment (characterized by high metabolic flux, endogenous ROS generation, and a reliance on redox‐active iron) that directly primes these cells for the phospholipid hydroperoxide accumulation and membrane failure that define ferroptosis, as will be detailed in the following sections.

### 2.2 Unique Vulnerability of Dopaminergic Neurons

Cell death is a highly regulated process essential for maintaining tissue homeostasis and organ function (Galluzzi et al. 2007). In the mature central nervous system, however, the predominantly postmitotic nature of neurons necessitates their long‐term survival to preserve established neural circuits. Indeed, while the elimination of superfluous neurons during development is crucial for shaping functional circuitry (Fricker et al. 2018), aberrant neuronal death, along with neuronal dysfunction, in the mature nervous system is a primary driver of neurodegenerative diseases (Moujalled et al. 2021). Although multiple neuronal populations, including cholinergic, glutamatergic, serotonergic, and GABAergic neurons, are affected across the spectrum of neurodegenerative disorders (Azmitia and Nixon 2008; Bi et al. 2020; Cacabelos et al. 1999), dopaminergic neurons exhibit a unique biochemical profile that confers markedly increased susceptibility to degenerative stimuli. Notably, their vulnerability is not uniform, but follows a distinct topographical gradient: those within the substantia nigra pars compacta (SNpc) are more susceptible to degeneration than those in the adjacent ventral tegmental area (VTA). Furthermore, susceptibility is stratified even within the SNpc itself, with neurons of the ventral tier demonstrating profoundly greater vulnerability compared to their counterparts in the dorsal tier (Double et al. 2010). We next examine the characteristics that render specific dopaminergic neurons highly susceptible to degeneration.

#### 2.2.1 Electrophysiological Patterns, Ca^2+^ Buffering, and Metabolic Rate

Three highly interconnected cellular attributes (electrophysiological patterns, Ca^2+^ buffering demand, and high metabolic rate) collectively enhance the susceptibility of dopaminergic neurons to neurodegeneration. A key vulnerability factor is their unique electrophysiological profile, characterized by the ability to generate regular, spontaneous action potentials without synaptic input or other external triggers, a process known as pacemaking activity (Sulzer and Schmitz 2007). Critically, adult SNpc dopaminergic neurons rely on Ca^2+^ influx through Ca_v_1.3 L‐type channels to sustain this pacemaking (Chan et al. 2007). This dependence results in continuous and substantial Ca^2+^ influx, creating a significant buffering demand. To maintain cytosolic Ca^2+^ homeostasis, ATP‐dependent pumps must constantly sequester Ca^2+^ into the endoplasmic reticulum (ER) and mitochondria or export it from the cell (Berridge 2002; Choi et al. 2006). The high metabolic cost of this continuous ion pumping necessitates a sustained elevated metabolic rate in these neurons. This, in turn, increases mitochondrial electron transport chain activity and elevates the production of reactive oxygen species (Guzman et al. 2010; Ludtmann and Abramov 2018). The direct link between this Ca^2+^‐dependent pacemaking and vulnerability is evidenced by the finding that blocking Ca_v_1.3 channels is protective in both in vitro and in vivo models of PD (Chan et al. 2007). The crucial role of Ca^2+^ buffering capacity in determining neuronal vulnerability is further underscored by the presence of calbindin‐D28k, which is found in midbrain dopaminergic neurons that are less susceptible to degeneration in both PD and its animal models (German et al. 1992).

#### 2.2.2 High Iron Levels

The aforementioned high metabolic demand of dopaminergic neurons of the SNpc necessitates robust ATP synthesis, a process for which iron is indispensable. Iron serves as a crucial cofactor for mitochondrial iron–sulfur (Fe–S) clusters (Read et al. 2021) and cytochromes within the electron transport chain (Xu et al. 2013), and is also a required cofactor for tyrosine hydroxylase (Ramsey et al. 1996), the rate‐limiting enzyme in dopamine synthesis. Consequently, the high iron content in these neurons is fundamentally linked to their physiological function (Dev and Babitt 2017).

However, this iron enrichment becomes a significant vulnerability over time. Notably, iron levels increase within the aging brain in specific regions, including the SNpc, putamen, globus pallidus, and red nucleus (Bilgic et al. 2012). This accumulation is markedly pronounced in the SNpc of patients with PD, with postmortem analyses revealing a 31%–35% increase in total iron content compared to control tissue (Dexter, Wells, et al. 1989). However, it is important to note that elevated iron levels alone do not fully account for cellular susceptibility to degeneration. This is evident from the fact that oligodendrocytes and astrocytes contain higher iron concentrations than neurons (Reinert et al. 2019), yet are not similarly prone to degeneration. This observation aligns with the understanding that iron sequestered into ferritin, the cell's primary iron storage protein, is less toxic than redox‐active free iron. Thus, while increased total iron undoubtedly contributes to dopaminergic neuron death, additional factors modulate this vulnerability. These factors, which influence the levels of toxic free iron (e.g., ferritin), mitigate iron‐driven oxidative damage (e.g., antioxidant enzymes), or synergistically enhance iron‐mediated pro‐oxidative stress (e.g., dopamine), represent additional players that increase the susceptibility of dopaminergic neurons to degenerate.

The copresence of high iron and dopamine creates a unique chemical environment that is inherently risky. Indeed, an iron‐dopamine index appears to predict the risk of parkinsonian neurodegeneration in the SNpc. It is hypothesized that iron dyshomeostasis in PD disrupts chaperoning mechanisms, allowing iron to interact with dopamine and lead to the generation of cytotoxic reactive oxygen species (ROS) (Hare et al. 2014). This event is a potent source of oxidative stress, which is evidenced in PD by enhanced basal lipid peroxidation in the SNpc (Jenner et al. 1992). The disturbance of this delicate balance renders SNpc neurons exceptionally vulnerable to parkinsonian neurodegeneration.

This vulnerability is critically combined by a concomitant failure of antioxidant defenses, as discussed in the following subsection. In summary, the high iron content in nigral dopaminergic neurons is a double‐edged sword: It is crucial for supporting their specialized metabolic and neurochemical functions, yet it simultaneously elevates their susceptibility to oxidative damage and neurodegeneration. Nevertheless, additional factors modulate iron‐mediated toxicity by altering levels of free redox‐active iron and mitigating the resultant oxidative damage.

#### 2.2.3 Dopamine Metabolism, Oxidative Stress, and Compromised Antioxidant Defenses

Dopamine homeostasis is a tightly regulated process dependent on several key mechanisms: (i) de novo synthesis, governed primarily by the rate‐limiting enzyme tyrosine hydroxylase; (ii) vesicular sequestration by the vesicular monoamine transporter 2 (VMAT2), which minimizes cytotoxic cytosolic accumulation; and (iii) reuptake from the synaptic cleft via the dopamine transporter (DAT) for subsequent repackaging or enzymatic degradation (Meiser et al. 2013). The elevated vulnerability of dopaminergic neurons to degeneration, compared to other neuronal populations, is intrinsically linked to dopamine metabolism. A primary source of this vulnerability is the cytosolic accumulation of dopamine, which occurs when VMAT2‐mediated sequestration is low; there is an inverse relationship between VMAT2 levels and the vulnerability to degeneration in PD (Meiser et al. 2013). Supporting this, isolated striatal dopamine storage vesicles from PD patients exhibit diminished vesicular uptake capacity. This finding implicates VMAT2 dysfunction as a direct cause of impaired dopamine storage and suggests that the resulting elevated cytosolic dopamine contributes to neurodegeneration (Pifl et al. 2014).

Once in the cytosol, dopamine is metabolized through enzymatic pathways, as well as nonenzymatic auto‐oxidation. Both pathways are potent generators of reactive oxygen species (ROS), including hydrogen peroxide, superoxide anion, and highly toxic hydroxyl radicals (Barzilai et al. 2001). Furthermore, MAO metabolism produces the toxic metabolite 3,4‐dihydroxyphenylacetaldehyde (DOPAL), which can inhibit its detoxifying enzyme, aldehyde dehydrogenase (ALDH1A1). This inhibition creates a vicious cycle of DOPAL accumulation that perpetuates oxidative stress and promotes lipid peroxidation (Masato et al. 2019). Concurrently, dopamine auto‐oxidation generates reactive quinones that form cytotoxic adducts, inhibiting crucial cellular functions such as proteasomal activity and parkin ligase function, and damaging essential macromolecules (LaVoie et al. 2005; Zafar et al. 2006). This prooxidant intracellular environment significantly contributes to dopaminergic cell death.

The susceptibility of these neurons is not solely due to heightened ROS generation but is critically intensified by a concomitant decline in antioxidant defense mechanisms. Even in healthy aging, a progressive, age‐dependent reduction in the function and levels of key antioxidants occurs alongside rising ROS production in the SNpc. Postmortem analyses show a region‐specific decrease in total glutathione (reduced + oxidized forms), as well as reduced activities of total superoxide dismutase (SOD), total glutathione peroxidase (GPx), and glutathione reductase in the SNpc of aged individuals compared to younger subjects (Venkateshappa et al. 2012). This indicates a gradual diminution in the capacity of nigral neurons to mitigate oxidative stress, thereby heightening regional vulnerability with age.

In the pathological context of PD, the antioxidant decline is severe and widespread, intensifying disease‐specific ROS generation. A hallmark of this failure is a drastic reduction (~50%) in both total glutathione levels and GPx activity, reflecting a profound dysfunction of the primary glutathione/GPx system (Sian et al. 1994), or their consumption by increasing oxidative stress. Critically, deficits in the glutathione/GPx system are already present in incidental Lewy body disease (Zeevalk et al. 2008), a pathological state considered to represent preclinical PD (DelleDonne et al. 2008). This suggests that antioxidant failure precedes overt neurodegeneration and may play a causative role in disease etiology. Although the mechanisms driving this significant antioxidant decrease remain incompletely understood, the aforementioned evidence establishes its central role in the cell death process, making it a major contributor to the susceptibility of dopaminergic neurons.

#### 2.2.4 Neuromelanin

The dopaminergic neurons of the SNpc are the most heavily pigmented cells in the human brain, accumulating the dark catecholamine‐derived pigment neuromelanin (Zucca et al. 2014). This pigment appears to be central to both neuronal maintenance and vulnerability, serving a protective role in the healthy brain by sequestering toxic compounds, including excess iron (Filimontseva et al. 2025; Zecca et al. 2008). In pathological contexts, however, this protective function can be subverted. In vivo models demonstrate that experimentally induced neuromelanin upregulation is sufficient to induce PD‐like motor deficits and neuropathology (Chocarro et al. 2023; Garcia‐Gomara et al. 2025). Furthermore, in vitro studies using dopaminergic neurons differentiated from PD patient‐derived iPSCs with DJ‐1 mutations show that neuromelanin accumulation coincides with elevated levels of oxidized dopamine (Burbulla et al. 2017). These lines of evidence position neuromelanin at a crucial intersection between physiological protection and pathological trigger (Filimontseva et al. 2025), establishing it as a significant factor determining the selective vulnerability of dopaminergic neurons to degeneration.

As detailed throughout this section, the convergence of several intrinsic factors, including a specific electrophysiological profile, elevated Ca^2+^ buffering demands, high basal metabolic rate, age‐ and disease‐related iron accumulation, dopamine metabolism, oxidative stress, and compromised antioxidant defenses, creates a unique combination of risk factors that, acting synergistically, drives chronic oxidative stress. This multifactorial interplay is central to the distinctive vulnerability of dopaminergic neurons and significantly contributes to the etiology of specific neurodegenerative diseases, most notably PD.

## 3 Iron, Ferroptosis, and Dopaminergic Neurodegeneration

The unique physiology of dopaminergic neurons renders them highly susceptible to neurodegeneration, with oxidative stress being a central mechanism. A key driver of this prooxidant environment is labile iron. While essential for metabolic and neurochemical functions, dysregulation of iron homeostasis significantly increases the vulnerability of these neurons to oxidative damage and subsequent degeneration.

The critical role of iron in neuronal toxicity is relevant to ferroptosis, an iron‐dependent form of regulated cell death. This connection is particularly important, given the well‐documented accumulation of iron in the dopaminergic brain regions affected in Parkinson's disease (PD), neuroferritinopathy, dementia with Lewy bodies, and related disorders. We examine next the molecular mechanisms of iron homeostasis and its central role in ferroptosis and dopaminergic degeneration.

### 3.1 Iron Homeostasis: Systemic and Cerebral Regulation

Iron is an essential nutrient with crucial roles in a broad array of biological processes, including oxygen transport, energy metabolism, DNA synthesis, and xenobiotic metabolism (Rouault 2006). Its utility stems from its ability to cycle between ferrous (Fe^2+^) and ferric (Fe^3+^) states, making it a prime cofactor for electron transfer in vital enzymes and proteins. Consequently, iron deficiency is associated with myriad systemic and neurological symptoms, including fatigue, impaired cognitive function, and restless legs syndrome (Auerbach et al. 2025). Conversely, excess iron is potently toxic due to its ability to catalyze, via the Fenton reaction, the production of highly reactive oxidant species such as the hydroxyl radical. This leads to deleterious oxidation of biomolecules including DNA, proteins, lipids, and small molecules like ascorbate and biogenic amines (Welch et al. 2002). Therefore, under physiological conditions, systemic and cellular iron homeostasis is exquisitely and tightly regulated (Andrews and Schmidt 2007). This regulation operates on multiple levels: from whole‐body absorption and distribution to cellular import, storage, export, and posttranscriptional control. The brain presents a unique compartment with specialized barriers and cell‐specific mechanisms, making its iron homeostasis particularly complex and vulnerable to dysregulation, which is a hallmark of several neurodegenerative conditions. Below, we provide an overview of the mechanisms mediating systemic and cerebral iron homeostasis, establishing the foundation for understanding its role in subsequent pathological processes like lipid peroxidation.

#### 3.1.1 Systemic Iron Balance: Absorption and Hormonal Control

The total body iron content is primarily determined by the regulation of dietary iron absorption in the duodenum and proximal jejunum, as there is no active excretory pathway for excess iron. Nonheme dietary iron, predominantly in the ferric (Fe^3+^) form, must be reduced to ferrous (Fe^2+^) iron by brush‐border ferrireductases like duodenal cytochrome B before it can be transported into enterocytes by divalent metal transporter 1 (DMT1) (McKie et al. 2001; Fuqua et al. 2012). Within the enterocyte, iron can be stored in ferritin or exported across the basolateral membrane into the circulation via ferroportin (FPN1), the sole known mammalian cellular iron exporter (Donovan et al. 2000; Gunshin et al. 1997). This exported Fe^2+^ is immediately oxidized to Fe^3+^ by the ferroxidase hephaestin, enabling its loading onto the circulating glycoprotein transferrin (Vulpe et al. 1999).

This export process is the master regulatory node for systemic iron, governed by the hepatic peptide hormone hepcidin (Ganz 2008). When body iron stores are sufficient or during inflammation, hepcidin is upregulated. It binds to ferroportin on enterocytes, macrophages, and hepatocytes, inducing its internalization and degradation, thereby blocking iron efflux into the plasma and sequestering iron within cells (Ganz 2008; Słomka et al. 2015). This hormonal regulation ensures that systemic iron availability matches demand, preventing both deficiency and overload. Dysregulation of this axis, as in hereditary hemochromatosis where hepcidin is deficient, leads to unbridled iron absorption and systemic iron overload, resulting in oxidative damage to organs like the liver, pancreas, and heart (Hentze et al. 2010).

#### 3.1.2 Cerebral Iron Homeostasis: Crossing and Navigating the Brain

The brain's high metabolic demand requires substantial iron for processes like oxidative phosphorylation, myelination, and neurotransmitter synthesis (Beard and Connor 2003). However, its iron homeostasis is uniquely constrained by the blood–brain barrier (BBB) and blood‐cerebrospinal fluid barrier and is managed by a concerted interplay between endothelial cells, astrocytes, microglia, oligodendrocytes, and neurons.

##### 3.1.2.1 Import Across the BBB

Under physiological conditions, the dominant pathway for brain iron acquisition is receptor‐mediated transcytosis of transferrin‐bound iron (Tf‐Fe). Holotransferrin binds to transferrin receptor 1 (TfR1) on the luminal surface of brain capillary endothelial cells (BCECs). The complex is endocytosed, and within acidified endosomes, Fe^3+^ is released, reduced to Fe^2+^ by reductases like STEAP3, and transported into the endothelial cytosol via DMT1 (Kawabata 2019; Reichert et al. 2020). From the cytosol, iron is exported across the abluminal membrane into the brain interstitial fluid via ferroportin. The ferroxidase ceruloplasmin, abundantly present in the interstitial space, re‐oxidizes Fe^2+^ to Fe^3+^, facilitating its rebinding to apotransferrin to form holotransferrin available for parenchymal cells (Reichert et al. 2020). While non‐transferrin‐bound iron (NTBI) is a minor component in plasma, it may become relevant in conditions of iron overload or barrier dysfunction and can enter BCECs and brain cells via transporters such as ZIP14, ZIP8, and DMT1 (Knutson 2019; Ji and Kosman 2015).

##### 3.1.2.2 Cellular Iron Handling in the Brain Parenchyma

Once in the interstitial fluid, iron is distributed to neural cells via specific mechanisms: Neurons primarily acquire iron via TfR1‐mediated endocytosis of transferrin, but also express surface DMT1 and ZIP8 for NTBI uptake (Ji and Kosman 2015). Oligodendrocytes, essential for myelination, are highly iron‐dependent. They express TfR1 and also utilize specific receptors like Tim‐2 to take up iron‐loaded H‐ferritin, a crucial mechanism for their iron supply (Todorich et al. 2011, 2008). Astrocytes and microglia play key supportive and regulatory roles. They express TfR1 and DMT1 for iron uptake and are critically involved in iron buffering, storage, and release, influencing neuronal iron availability (Lis et al. 2004; Urrutia et al. 2013).

Within all cells, cytosolic iron enters a chelatable, metabolically active labile iron pool (LIP). To prevent oxidative damage, excess Fe^2+^ is rapidly sequestered by ferritin, a multimeric protein composed of heavy (FTH1) and light (FTL) chains. FTH1 possesses ferroxidase activity, converting Fe^2+^ to less‐reactive Fe^3+^ for safe mineralization within its core (Knutson 2008; Kotla et al. 2022). Notably, degradation of ferritin via ferritinophagy, mediated by the cargo receptor NCOA4, releases iron into the LIP, favoring iron‐mediated cell damage (Yoshida et al. 2019; Li et al. 2020). Conversely, exosome‐mediated export of ferritin by cells under stress is an anti‐ferroptotic adaptation (Brown et al. 2019). The embryonic lethality of FTH1 knockout mice underscores ferritin's essential cytoprotective role (Knovich et al. 2009). Mitochondria, major sites of iron utilization for heme and iron–sulfur cluster synthesis, possess their own unique storage protein, mitochondrial ferritin (FtMt), which is thought to protect these organelles from iron‐mediated oxidative stress (Levi et al. 2001; Santambrogio et al. 2007), Cellular iron export from neurons and glia, as elsewhere, is mediated by ferroportin, subject to regulation by local and systemic signals.

#### 3.1.3 Fine‐Tuning: The Hepcidin and IRE/IRP Regulatory Systems in the Brain

Cerebral iron homeostasis is finely tuned by two major regulatory systems that respond to local iron levels and inflammatory cues.

##### 3.1.3.1 The Hepcidin‐Ferroportin Axis in the CNS

Hepcidin is not only a systemic hormone but is also produced within the brain by neurons, astrocytes, and microglia in response to iron and inflammation (Wang et al. 2008; Qian et al. 2014). Its local expression is regulated by pathways analogous to the liver, including the IL‐6/JAK2/STAT3 and BMP/SMAD pathways (Qian et al. 2014; Silvestri et al. 2019). Under physiological conditions, brain‐derived hepcidin likely acts in a paracrine/autocrine manner to inhibit neuronal and glial ferroportin, modulating regional iron distribution (Vela 2018). However, under inflammatory conditions, triggered by factors like lipopolysaccharide (LPS) or hemorrhage, hepcidin is markedly upregulated via Toll‐like receptor (TLR) signaling (Xiong et al. 2016; You et al. 2017). This leads to excessive ferroportin internalization, trapping iron inside neurons and glia, elevating the LIP, and precipitating oxidative damage and apoptosis (Urrutia et al. 2013; Zhou et al. 2017). This mechanism links neuroinflammation directly to iron dyshomeostasis and neuronal vulnerability.

##### 3.1.3.2 The Iron‐Responsive Element/Iron Regulatory Protein (IRE/IRP) System

At the posttranscriptional level, cellular iron balance is governed by the IRE/IRP system, a sensitive rheostat that coordinates the expression of key iron metabolism proteins based on cytosolic iron availability (Hentze et al. 2010). When iron is scarce, iron regulatory proteins 1 and 2 (IRP1, IRP2) bind with high affinity to conserved hairpin‐loop sequences called IREs in the untranslated regions (UTRs) of target mRNAs. This binding stabilizes mRNAs for proteins involved in iron acquisition (e.g., TfR1, DMT1) by preventing their degradation, while simultaneously blocking the translation of mRNAs for iron storage and export proteins (e.g., ferritin, ferroportin) (Muckenthaler et al. 1998; Abboud and Haile 2000). When iron is abundant, IRPs lose their RNA‐binding activity, leading to decreased TfR1/DMT1 synthesis and increased production of ferritin and ferroportin, thereby promoting safe storage and efflux. This system ensures that cellular iron uptake, storage, and export are dynamically and coordinately regulated.

**FIGURE 1:**
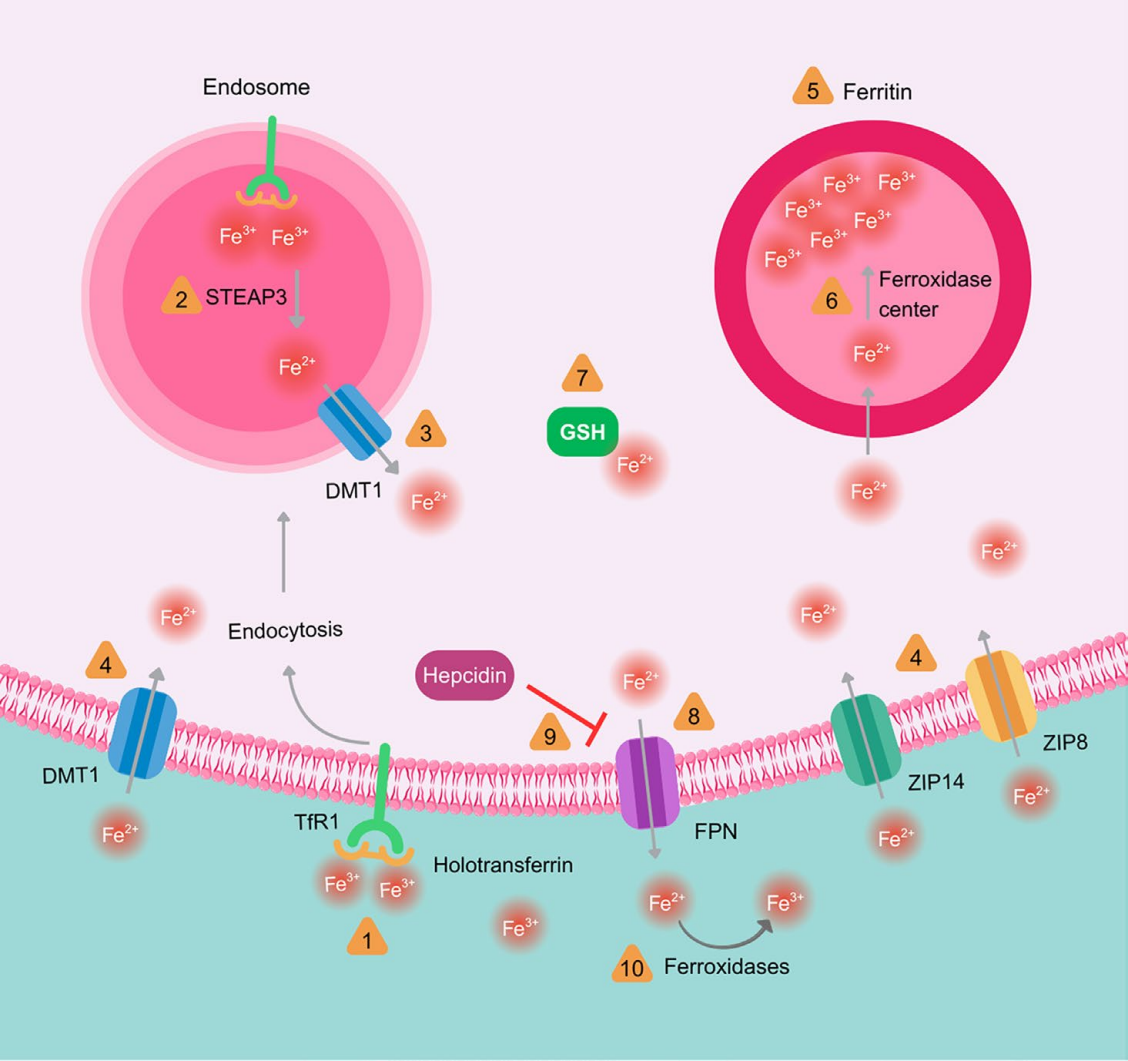
Major events and mechanisms mediating iron homeostasis. The primary route for cellular iron entry is the transferrin (Tf)‐dependent pathway. Holotransferrin binds to transferrin receptor 1 (TfR1) on the cell surface, and the complex is internalized via endocytosis (*event 1*). Within acidified endosomes, ferric iron (Fe^3+^) is released from transferrin and reduced to ferrous iron (Fe^2+^) by metalloreductases such as six‐transmembrane epithelial antigen of prostate 3 (STEAP3) (*event 2*). This Fe^2+^ can then be transported into the cytosol by divalent metal transporter 1 (DMT1) (*event 3*). Iron can also enter cells as non‐transferrin‐bound iron (NTBI) via transporters including ZIP14, ZIP8, and DMT1 (*event 4*). In the cytosol, excess Fe^2+^ is sequestered by ferritin, the major iron storage protein (*event 5*). Ferritin's ferroxidase activity oxidizes Fe^2+^ to Fe^3+^ for safe storage within its mineral core (*event 6*). Cytosolic Fe^2+^ can also be chelated by glutathione (GSH) (*event 7*). Excess cellular iron is exported via ferroportin (FPN) (*event 8*), the sole mammalian iron exporter, whose activity is inhibited by the hormone hepcidin (*event 9*). The exported Fe^2+^ is oxidized to Fe^3+^ by ferroxidases like ceruloplasmin and hephaestin (*event 10*). Events 1–10 are indicated as orange triangles. References for the aforementioned events are present in the main text.

The major events and mechanisms mediating iron homeostasis are depicted in Figure 1.

#### 3.1.4 Integration and Pathophysiological Implications

The complex, multilayered interplay between barrier transport, cellular uptake/storage/export, and the hepcidin and IRE/IRP regulatory systems is the product of evolution, finely tuned to harness iron's essential biochemical properties while mitigating its inherent toxicity. The critical importance of this buffering capacity is starkly demonstrated not only by the systemic pathology of hemochromatosis but also by neurodegenerative disorders such as PD and others associated with brain iron accumulation (McNally et al. 2019).

It is crucial to recognize that total iron concentration alone is a poor predictor of toxicity. As evidenced by the fact that oligodendrocytes and astrocytes routinely maintain higher iron levels than neurons without undergoing degeneration, the speciation and compartmentalization of iron are paramount. Iron safely sequestered within ferritin's mineral core is redox‐inert. The primary toxic species is the redox‐active LIP, consisting of loosely chelated Fe^2+^ capable of participating in Fenton chemistry. Therefore, the vulnerability of a cell like a dopaminergic neuron to iron‐mediated degeneration is not determined merely by its iron content, but by the dynamic balance between prooxidant forces (e.g., high LIP, presence of dopamine autoxidation products) and antioxidant/cytoprotective defenses (e.g., ferritin buffering capacity, glutathione, antioxidant enzymes).

This framework establishes that iron homeostasis is a dynamic, multi‐compartmental process. Its dysregulation, whether through increased influx, impaired storage, deficient export, or inflammatory signaling, can lead to a pathological expansion of the LIP. This sets the stage for the rampant oxidation of cellular constituents. Among these, the peroxidation of lipids, given their abundance in brain membranes and their susceptibility to radical chain reactions, emerges as a particularly devastating consequence, directly linking iron dyshomeostasis to the loss of neuronal integrity and function, as discussed in the following sections.

#### 3.1.5 Iron and Lipid Peroxidation

Lipid peroxidation is a self‐propagating chain reaction in which oxidant species, particularly oxygen free radicals, attack the polyunsaturated fatty acid (PUFA) tails of membrane phospholipids. This oxidation generates lipid peroxides, leading to cellular damage (Ayala et al. 2014). Lipid peroxidation can be initiated in cells by nonenzymatic and enzymatic mechanisms. The nonenzymatic pathway is mediated by redox‐active metals, particularly iron. Within cells, labile iron can react with endogenously produced hydrogen peroxide (H_2_O_2_) via the Fenton reaction (Fe^2+^ + H_2_O_2_ → Fe^3+^ + HO˙ + OH^−^) to generate the highly reactive hydroxyl radical (HO˙) (Lai and Piette 1978). The hydroxyl radical can then abstract a hydrogen atom from a neighboring membrane lipid (L, commonly a PUFA of membrane phospholipids), generating a carbon‐centered lipid radical (L˙). In a propagation phase, L˙ reacts with molecular oxygen (O_2_) to form a lipid peroxyl radical (LOO˙), which can abstract one hydrogen (H˙) from another neighboring lipid, generating another lipid radical and a hydroperoxide (LOOH) in a membrane phospholipid hydroperoxide (PLOOH). Like H_2_O_2_, PLOOH can undergo an iron‐catalyzed reaction (with Fe^2+^), generating a phospholipid alkoxyl radical (PLO˙). This radical can, in turn, abstract one hydrogen from a neighboring PUFA‐containing phospholipid, thereby propagating the peroxidation chain reaction. If not rapidly neutralized (e.g., by glutathione peroxidase 4 (GPx4)), this chain reaction can extensively damage cell membranes. Although the Fenton reaction is well‐characterized in vitro, its role in cells and tissues is not fully elucidated, and it may be significant primarily in severe iron overload states or other pathological conditions with disturbed iron metabolism (Stoyanovsky et al. 2019; Zheng et al. 2024).

In addition to its role in nonenzymatic peroxidation via radical generation, iron is an essential cofactor for lipoxygenases (LOXs). This family of iron‐containing enzymes catalyzes the stereospecific dioxygenation of free PUFAs to produce fatty acid hydroperoxides (Haeggström and Funk 2011), and of PUFA‐phospholipids when complexed with PEBP1 (Wenzel et al. 2017). Notably, PUFA and PUFA‐PL oxidation by lipoxygenases and their complexes with PEBP1 has been implicated in cell death under specific conditions, and evidence indicates that lipoxygenase activity can stimulate ferroptosis (Yang et al. 2016). Notably, lipid peroxidation is a driver event of ferroptosis, discussed in the next section.

### 3.2 Ferroptosis—An Overview

Ferroptosis is an iron‐dependent, regulated form of necrotic cell death characterized by the overwhelming peroxidation of phospholipids (PLs) containing polyunsaturated fatty acids (PUFAs) in cellular membranes (Dixon et al. 2012; Stockwell 2022). The term was first introduced in 2012, but its biochemical and genetic foundations were established through decades of prior research on lipid peroxidation, glutathione metabolism, and selenium biology (Dixon et al. 2012; Hirschhorn and Stockwell 2019). Since its formal definition, ferroptosis has been implicated in a wide spectrum of pathological conditions, including acute and chronic organ injury (e.g., kidney, liver, heart, brain), cancer, neurodegeneration, autoimmune diseases, and immune dysregulation (Friedmann Angeli et al. 2014; Qi et al. 2020; Shen et al. 2022).

Morphologically, ferroptosis is distinct from apoptosis, necroptosis, and autophagy. It does not feature chromatin condensation, apoptotic body formation, or autophagic vacuoles. Instead, its ultrastructural hallmarks include mitochondrial pathology, specifically, mitochondrial shrinkage, increased membrane density, reduction or disappearance of cristae, and outer membrane rupture, along with a generally intact nucleus (Dalseno et al. 2025; Reichert et al. 2020). Biochemically, the core hallmarks of ferroptosis include the excessive, iron‐driven accumulation of phospholipid hydroperoxides (PLOOH), ultimately leading to plasma membrane permeabilization and cell death, alongside the characteristic depletion of glutathione (GSH) and inactivation or loss of glutathione peroxidase 4 (GPx4) (Jiang et al. 2021).

The execution of ferroptosis is governed by the interplay of three fundamental determinants: the availability of redox‐active (labile) iron, the abundance of oxidizable PUFA‐containing phospholipid substrates in cellular membranes, and the functional capacity of cellular antioxidant defense systems. The following sections provide a detailed synthesis of these core mechanisms, integrating recent advances that highlight the complexity and therapeutic relevance of ferroptosis.

#### 3.2.1 The Central Role of Redox‐Active Iron in Ferroptosis

The prefix “ferro‐,” from the Latin *ferrum* for iron, underscores the indispensable catalytic role of intracellular iron in this cell death pathway. The efficacy of iron chelators such as deferoxamine in inhibiting ferroptosis provides definitive proof of the metal's critical function (Dixon et al. 2012). Iron contributes to ferroptosis primarily through two interconnected mechanisms: catalyzing the nonenzymatic initiation and propagation of lipid peroxidation via Fenton/Fenton‐like chemistry and serving as an essential cofactor for enzymes like lipoxygenases (LOXs) that can enzymatically generate lipid peroxides (Yang et al. 2016).

##### 3.2.1.1 The Fenton Reaction and Lipid Peroxidation

In the Fenton reaction, ferrous iron (Fe^2+^) reacts with hydrogen peroxide (H_2_O_2_) to generate the highly reactive hydroxyl radical (HO˙) (Lai and Piette 1978). This radical can abstract a hydrogen atom from a bis‐allylic carbon in a PUFA, initiating a free‐radical chain reaction of lipid peroxidation. Furthermore, Fe^2+^ can directly react with preexisting lipid hydroperoxides (LOOH) in a Fenton‐like reaction to produce lipid alkoxyl radicals (LO˙), which are also potent initiators of new peroxidation cycles. This creates an auto‐amplifying loop, as the products of lipid peroxidation (LOOH) themselves generate more radicals in the presence of iron.

It is important to mention that the size and reactivity of the cellular LIP, which is a key determinant of ferroptosis susceptibility, is regulated by a network of proteins controlling iron uptake (i.e., TfR1, ZIP14), storage (ferritin), and export (FPN1, hepcidin), already discussed in Sections 3.1.1 and 3.1.2.

In addition to cytosolic iron, distinct subcellular iron pools are relevant. Lysosomal iron, often accumulated via endocytosis of iron‐rich proteins, is a potent driver of ferroptosis, as exemplified by the mechanism of the natural product ironomycin (Mai et al. 2017) and the synthetic compound fentomycin (Cañeque et al. 2025). Mitochondrial iron homeostasis, regulated by proteins like mitoferrins and CISD1/2 (mitoNEET/NAF1), also modulates ferroptotic sensitivity, highlighting the organelle‐specific roles of iron in this process (Fang et al. 2023).

#### 3.2.2 PUFAs Availability and Lipid Metabolism in Cell Membranes

The specific lipid composition of cellular membranes is a primary determinant of ferroptosis susceptibility. This form of cell death is fundamentally dependent on phospholipids containing PUFAs (PUFA‐PLs), which possess bis‐allylic methylene groups that are highly vulnerable to hydrogen abstraction by radicals. The presence of multiple double bonds allows the formation of resonance‐stabilized carbon‐centered radicals that rapidly react with oxygen, propagating the peroxidation chain reaction (Qiu et al. 2024).

##### 3.2.2.1 Key Lipid Species Driving Ferroptosis

Recent research has identified specific phospholipid species as major ferroptosis drivers. Phosphatidylethanolamines (PEs) containing arachidonic acid (AA, C20:4) or adrenic acid (AdA, C22:4) are particularly susceptible (Kagan et al. 2017). Strikingly, phospholipids with two PUFA tails (diPUFA‐PLs), such as C20:4/C20:4‐PC, have been identified as key proximal mediators of ferroptosis due to their extreme oxidizability (Qiu et al. 2024). Ether‐linked phospholipids (plasmalogens) containing PUFAs at the sn‐2 position are also highly prone to peroxidation (Zou et al. 2020).

##### 3.2.2.2 The Protective Role of Monounsaturated Fatty Acids (MUFAs)

In direct opposition to PUFAs, phospholipids containing MUFAs, such as oleic acid (C18:1), confer robust resistance to ferroptosis (Magtanong et al. 2019; Mann et al. 2024; Yang et al. 2016). The incorporation of MUFAs into membrane PLs dilutes the concentration of oxidizable PUFAs and may also alter membrane biophysical properties. Cells exposed to a MUFA‐rich microenvironment can remodel their membrane lipidome via ACSL3 activity to incorporate oleate, thereby gaining a survival advantage (Magtanong et al. 2019).

##### 3.2.2.3 Enzymatic Regulation of the Lipidome

The cellular lipidome is dynamically shaped by specific enzymes that channel fatty acids into phospholipids, creating a balance between pro‐ and anti‐ferroptotic states. The pro‐ferroptotic axis (ACSL4/LPCAT3) favors the biosynthesis of pro‐ferroptotic PUFA‐PLs; it is initiated by acyl‐CoA synthetase long‐chain family member 4 (ACSL4), which preferentially activates long‐chain PUFAs like AA and AdA (Yuan et al. 2016; Doll et al. 2017). ACSL4 expression and activity are therefore strong predictors of ferroptosis sensitivity. Its activity can be enhanced by PKCβII‐mediated phosphorylation, creating a positive feedback loop that amplifies ferroptosis (Zhang et al. 2022; Rodencal and Dixon 2022). Subsequently, lysophosphatidylcholine acyltransferase 3 (LPCAT3) incorporates these ACSL4‐generated PUFA‐CoAs into membrane phospholipids, further promoting ferroptosis (Reed et al. 2022). The transcription factor ZEB1, a master regulator of epithelial‐mesenchymal transition, promotes ferroptosis by upregulating enzymes in this axis (Schwab et al. 2024). In the opposing direction, the anti‐ferroptotic axis (ACSL3/MBOAT1/2) is comprised of enzymes that promote MUFA incorporation and suppress ferroptosis. ACSL3 activates MUFAs like oleate for incorporation into PLs. The membrane‐bound O‐acyltransferases MBOAT1 and MBOAT2 preferentially utilize MUFA‐CoAs to re‐acylate lysophospholipids (e.g., lysophosphatidylethanolamine) during the Lands' cycle of phospholipid remodeling (Li, Hu, et al. 2024; Liang et al. 2023).

##### 3.2.2.4 Lipid Peroxidation Enzymes

Once incorporated into membranes, PUFA‐PLs can be peroxidized through nonenzymatic autoxidation (driven by Fenton chemistry) or via enzymatic catalysis. Iron‐containing arachidonate lipoxygenases (ALOXs, especially ALOX15/15‐LOX‐1) and cytochrome P450 oxidoreductase (POR) can contribute to the generation of specific lipid hydroperoxides that seed the ferroptosis cascade (Abeysinghe et al. 1996; Li, Wang, et al. 2023; Hadian and Stockwell 2023).

#### 3.2.3 Availability of Antioxidants

Cellular defense against ferroptosis is orchestrated by a multilayered network of enzymatic and nonenzymatic antioxidant systems. The failure of these systems, either through genetic disruption, pharmacological inhibition, or metabolic insufficiency, unleashes the lethal lipid peroxidation cascade.

##### 3.2.3.1 The Canonical System Xc−/GSH/GPx4 Axis

This is the primary and most well‐characterized anti‐ferroptotic pathway (Friedmann Angeli et al. 2014; Li et al. 2022). The system operates through a sequence of key biochemical events. It begins with cystine uptake, where the cystine/glutamate antiporter imports extracellular cystine while exporting glutamate. Inside the cell, cystine is rapidly reduced to cysteine. This cysteine serves as the critical rate‐limiting precursor for glutathione (GSH) synthesis, a two‐step process catalyzed by the enzymes γ‐glutamylcysteine ligase and glutathione synthase. Finally, the selenoenzyme GPx4 utilizes the synthesized GSH as a reducing cofactor. It directly neutralizes toxic lipid hydroperoxides, converting phospholipid and cholesterol hydroperoxides into their corresponding nontoxic alcohols. This action halts the destructive chain reaction of lipid peroxidation.

The centrality of this axis is demonstrated by the fact that its disruption at any point (using system xCT inhibitors [e.g., erastin, sulfasalazine], GSH synthesis inhibitors [e.g., buthionine sulfoximine, BSO], or direct covalent GPx4 inhibitors [e.g., RSL3, ML162]) is a standard method to induce ferroptosis (Stockwell and Jiang 2020); however, BSO is a weak ferroptosis inducer for reasons that are not well understood. GPx4 is essential for life; its complete knockout is embryonically lethal in mice, and its conditional deletion in various tissues (neurons, kidney, T cells, etc.) leads to rapid ferroptosis and organ failure (Yant et al. 2003; Seiler et al. 2008).

##### 3.2.3.2 The FSP1/Coenzyme Q_10_ Axis

A major breakthrough was the discovery of a powerful, GPx4‐independent ferroptosis suppression pathway centered on ferroptosis suppressor protein 1 (FSP1, formerly AIFM2) and coenzyme Q_10_ (CoQ_10_) (Bersuker et al. 2019; Doll et al. 2019). This pathway functions through the myristoylated, NAD(P)H‐dependent oxidoreductase FSP1, which is localized to the plasma membrane. FSP1 reduces oxidized ubiquinone (CoQ_10_) to its active, radical‐trapping hydroquinone form (ubiquinol, CoQ_10_H_2_). Acting as a potent lipophilic radical‐trapping antioxidant within the lipid bilayer, CoQ_10_H_2_ directly reduces lipid peroxyl radicals to lipid hydroperoxides, thereby halting the propagation of peroxidation chains.

The FSP1‐CoQ_10_ axis represents a crucial cellular backup system, and its expression correlates strongly with resistance to GPx4 inhibitors in many cancers. Consequently, the pharmacological inhibition of FSP1 (e.g., with iFSP1 or viFSP1) synergizes powerfully with GPx4 inhibition to trigger ferroptosis, establishing it as a highly promising combinatorial therapeutic strategy (Doll et al. 2019). In some cancers, FSP1 itself becomes a dependency, independent of GPx4, for unclear reasons (Wu et al. 2026; Palma et al. 2026).

##### 3.2.3.3 The GCH1/BH_4_/DHFR Axis—A Regulator of Endogenous Antioxidants

A distinct and independent ferroptosis suppression pathway operates through the pteridine tetrahydrobiopterin (BH_4_), whose synthesis is regulated by the rate‐limiting enzyme GTP cyclohydrolase 1 (GCH1) (Kraft et al. 2020). In this system, BH_4_ functions as a potent lipophilic radical‐trapping antioxidant by directly scavenging lipid radicals. Beyond its direct antioxidant role, the GCH1/BH_4_ axis also amplifies cellular protection by promoting the accumulation of reduced coenzyme Q_10_ (CoQ_10_H_2_), thereby reinforcing the FSP1/CoQ_10_ pathway. The oxidized form of BH_4_, BH_2_, is continuously recycled back to its active state by dihydrofolate reductase (DHFR), maintaining a protective pool.

##### 3.2.3.4 Lipophilic Vitamins as Endogenous Radical‐Trapping Antioxidants

Beyond dedicated enzymatic pathways, several lipophilic vitamins serve as essential endogenous radical‐trapping antioxidants. The premier example is vitamin E (α‐tocopherol), which neutralizes lipid peroxyl radicals by donating a hydrogen atom from its phenolic hydroxyl group. This action halts peroxidation propagation by forming a stable tocopheryl radical (Friedmann Angeli et al. 2014; Seiler et al. 2008; Niki 2021). Its physiological importance is underscored by evidence that dietary vitamin E can compensate for GPx4 loss in several tissues, while vitamin E deficiency is linked to neurodegeneration, a condition consistent with the failure to suppress neuronal ferroptosis (Carlson et al. 2016). Similarly, vitamin K functions as a potent radical‐trapping antioxidant when reduced to its hydroquinone form. Notably, this reduction is facilitated by FSP1 in a “noncanonical” cycle (Mishima et al. 2022).

##### 3.2.3.5 The KEAP1‐NRF2 System

Given that ferroptosis is fundamentally an oxidative cell death, it is unsurprising that the major transcriptional regulator of the antioxidant response, nuclear factor erythroid 2‐related factor 2 (NRF2), plays a pivotal role in modulating ferroptosis susceptibility. The KEAP1‐NRF2 system acts as a central hub integrating signals from oxidative stress to coordinate a broad cytoprotective program (Yamamoto et al. 2018), many elements of which directly counteract the drivers of ferroptosis.

Under basal conditions, NRF2 is bound by its negative regulator, Kelch‐like ECH‐associated protein 1 (KEAP1), which targets it for ubiquitination and proteasomal degradation. Electrophilic stressors or reactive oxygen species modify critical cysteine residues in KEAP1, inhibiting its E3 ligase activity. This leads to NRF2 stabilization, nuclear translocation, and binding to antioxidant response elements (AREs) in the promoter regions of hundreds of target genes (Zgorzynska et al. 2021).

The activation of NRF2 suppresses ferroptosis through a coordinated suite of molecular mechanisms. First, it enhances the core GSH/GPx4 axis by directly upregulating the genes involved in glutathione synthesis and metabolism. This includes the catalytic and modifier subunits of glutamate‐cysteine ligase (GCLC, GCLM) and glutathione reductase (GSR) (Cuadrado et al. 2009). Critically, NRF2 also transactivates SLC7A11 (Liu et al. 2025), the gene encoding the xCT subunit of the system xc‐ antiporter, thereby boosting cystine uptake and the cellular supply of GSH precursors, which directly supports the anti‐ferroptotic function of GPx4. A second key mechanism involves providing reducing equivalents. NRF2 upregulates enzymes of the pentose phosphate pathway and other NADPH‐producing pathways (Mitsuishi et al. 2012). The resulting elevation in cellular NADPH is essential for regenerating reduced glutathione and thioredoxin, and also serves as the vital electron donor for the FSP1/CoQ_10_ axis. By bolstering these antioxidant reductases, NRF2 sustains multiple defense systems against lipid peroxidation (Mitsuishi et al. 2012). Third, NRF2 exerts control over iron metabolism to limit Fenton chemistry. It transcriptionally upregulates ferritin heavy and light chains (FTH1, FTL) to sequester iron safely within its storage protein, and also promotes the expression of the iron exporter ferroportin (FPN1) (Agyeman et al. 2012; Harada et al. 2011). This combined action reduces the labile, redox‐active iron pool, thereby diminishing the primary catalyst for peroxidation chain reactions.

**FIGURE 2:**
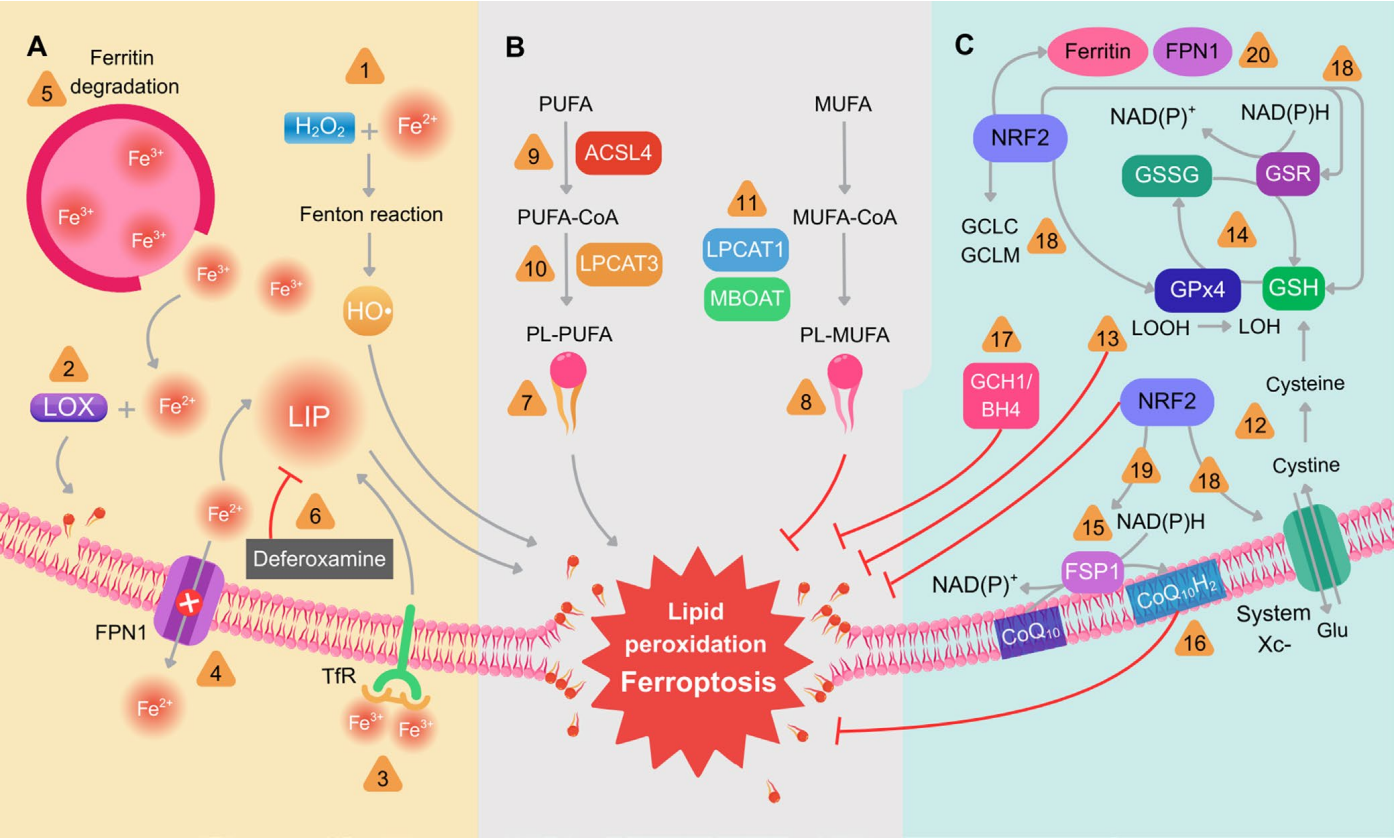
Major molecular events modulating ferroptosis. (A) The central role of redox‐active iron in ferroptosis. Iron's role in catalyzing lipid peroxidation through the Fenton reaction: labile iron generates the highly reactive hydroxyl radical (HO˙), which initiates nonenzymatic lipid peroxidation (*event 1*). Iron's role in catalyzing lipid peroxidation by acting as an essential cofactor for lipoxygenase (LOX) enzymes, which catalyze the enzymatic oxidation of lipids (*event 2*). Any process that increases the cellular labile iron pool (LIP), such as enhanced transferrin‐mediated uptake (*event 3*), inhibition of the ferroportin (FPN1) iron exporter (*event 4*), or degradation of ferritin (*event 5*), sensitizes cells to ferroptosis. The efficacy of iron chelators like deferoxamine in inhibiting this process highlights the metal's critical function (*event 6*). (B) PUFAs availability in cell membranes. Phospholipids containing polyunsaturated fatty acyl tails (PL‐PUFAs) are major drivers of ferroptosis because of their exceptional susceptibility to peroxidation (*event 7*). Conversely, monounsaturated fatty acids (MUFAs) incorporate into membrane phospholipids, displacing oxidizable PUFAs, suppressing lipid peroxidation and inhibiting ferroptosis (*event 8*). The biosynthesis of pro‐ferroptotic PUFA‐containing phospholipids is initiated by acyl‐CoA synthetase long‐chain member 4 (ACSL4), which activates free polyunsaturated fatty acyl‐coenzyme A (PUFA‐CoAs) (*event 9*), followed by lysophosphatidylcholine acyltransferase 3 (LPCAT3)‐mediated incorporation of these PUFA‐CoAs into membrane phospholipids, further promoting ferroptosis sensitivity (*event 10*). In opposition, enzymes like membrane‐bound glycerophospholipid O‐acyltransferase 1/2 (MBOAT1/2) and lysophosphatidylcholine acyltransferase 1 (LPCAT1), which catalyze the formation of MUFA‐containing phospholipids, promote ferroptosis resistance (*event 11*). (C) Availability of antioxidants. A primary defense against ferroptosis is the System Xc−/GSH/GPx4 axis, which imports cystine, the oxidized form of cysteine, into cells with a 1:1 counter‐transport of glutamate (Glu). Cysteine, derived from cystine reduction, is a key precursor for glutathione (GSH) synthesis (*event 12*). Glutathione peroxidase 4 (GPx4) then utilizes GSH as a cofactor to reduce toxic lipid hydroperoxides to harmless lipid alcohols (*event 13*), thereby directly halting the peroxidation chain reaction. This event generated oxidized glutathione (GSSG), which is reduced back to GSH at the expense of nicotinamide adenine dinucleotide phosphate (NADPH) in a reaction catalyzed by glutathione reductase (GSR) (*event 14*). Alongside this canonical glutathione‐based system, there is the endogenous antioxidant coenzyme Q_10_ (CoQ_10_) and the enzyme ferroptosis suppressor protein 1 (FSP1), which functions as an NAD(P)H‐dependent oxidoreductase that reduces CoQ_10_ to its active, hydroquinone form (CoQ_10_H_2_) at the plasma membrane (*event 15*). This regenerated CoQ_10_H_2_ acts as a lipophilic radical‐trapping antioxidant (RTA), effectively scavenging lipid peroxyl radicals (LOO˙) and thereby halting the propagation of lipid peroxidation (*event 16*). Appropriate levels of CoQ_10_ are also dependent on GTP cyclohydrolase‐1 (GCH1) and its derivative tetrahydrobiopterin (BH4). The GCH1‐BH_4_‐phospholipid axis is a master regulator of ferroptosis resistance, controlling endogenous BH_4_ production, CoQ_10_ abundance, and the peroxidation of specific phospholipids (*event 17*). NRF2 plays a pivotal role on ferroptosis resistance enhancing the GSH/GPx4 axis by upregulating genes involved in glutathione synthesis and cystine uptake, including *GCLC*, *GCLM*, *GSR*, and *SLC7A11*, thereby sustaining GPx4 activity (*event 18*). In parallel, NRF2 increases NADPH production via activation of the pentose phosphate pathway, supporting glutathione and thioredoxin regeneration and the FSP1/CoQ_10_ antioxidant system (*event 19*). Additionally, NRF2 limits iron‐driven lipid peroxidation by inducing ferritin (*FTH1*, *FTL*) and ferroportin (*FPN1*) (*event 20*), reducing the labile iron pool and Fenton chemistry. Events 1–20 are indicated as orange triangles. References for the aforementioned events are present in the main text. Fe^2+^, ferrous iron; Fe^3+^, ferric iron; GCLC, glutamate‐cysteine ligase catalytic subunit; GCLM, glutamate‐cysteine ligase modifier subunit; H_2_O_2_, hydrogen peroxide; LOH, lipid alcohols; LOOH, lipid hydroperoxides; MUFA‐CoA, monounsaturated fatty acyl‐coenzyme A; NAD(P)^+^, oxidized form of nicotinamide adenine dinucleotide phosphate; PL‐MUFA, phospholipids containing monounsaturated fatty acyl tails; System Xc−, cystine/glutamate antiporter; TfR, transferrin receptor.

Figure 2 depicts the major molecular events modulating ferroptosis.

When summarizing the cellular antioxidant network, it is important to note that defense against ferroptosis is not dependent on any single pathway. Instead, it is ensured by a robust, multilayered system. The canonical GPx4 system, the parallel FSP1/CoQ_10_ and GCH1/BH_4_ axes, lipophilic vitamins, and the overarching transcriptional control by NRF2 all work in concert to maintain membrane lipid integrity. The relative importance of each pathway varies by cell type, metabolic state, and disease context. Of particular relevance to dopaminergic neurodegeneration, evidence indicates that several key factors underpinning ferroptosis are altered not only in models of Parkinson's disease and related disorders but also in human disease. The following section discusses these findings.

### 3.3 Ferroptosis and Dopaminergic Neurodegeneration

Having discussed the vulnerability of dopaminergic neurons and the overall mechanisms of ferroptosis, this section will now integrate these concepts to examine ferroptosis's role in dopaminergic neurodegeneration. This pathway is implicated in multiple neurodegenerative disorders characterized by dopaminergic neurodegeneration, including PD, dementia with Lewy bodies, NBIAs, among others (Angelova et al. 2020; Lei et al. 2025; Muller and Leavitt 2014; Peng et al. 2025; Villalón‐García et al. 2023). The discussion prioritizes experimental and clinical data from PD, given the relative abundance of evidence, but also incorporates findings from NBIA to provide a broader perspective.

#### 3.3.1 Iron Dyshomeostasis

An important driver of ferroptosis is the loss of cellular iron homeostasis, a process critically relevant to dopaminergic neurodegeneration. The causal role of iron in ferroptosis is demonstrated by the ability of redox‐active, labile iron to potently catalyze the lipid peroxidation that defines this type of cell death (Lai and Piette 1978), and further supported by experimental models showing that iron chelators exert potent anti‐ferroptotic effects (Dixon et al. 2012). This link is particularly significant in PD, as well as related conditions such as and NBIA, which are characterized by a pronounced increase in iron levels within brain regions central to dopaminergic neurotransmission, including the substantia nigra pars compacta (SNpc) and other basal ganglia structures (Neumann et al. 2000). This region‐specific iron accumulation implies that dopaminergic neurons in these areas possess an inherent vulnerability to ferroptosis. Indeed, a growing body of literature substantiates a strong link between dopaminergic neurodegeneration and ferroptosis, with iron dyshomeostasis playing a central role. This pathological imbalance appears to stem from specific alterations in the proteins that regulate cellular iron metabolism, including those controlling iron import or export.

Experimental models of PD have been instrumental in delineating the mechanisms underlying this iron overload. A consistent finding is the upregulation of iron import proteins. For instance, in a murine model of MPTP intoxication, Salazar et al. (2008) demonstrated an increase in divalent metal transporter 1 (DMT1) expression in the ventral mesencephalon, concomitant with iron accumulation, oxidative stress, and dopaminergic cell loss. Notably, the same study observed increased expression of a specific DMT1 isoform (Nramp2/Slc11a2) in the substantia nigra of PD patients. Evidence also implicates the transferrin receptor (TfR) system. In a murine PD model involving exposure to MPTP and the pyrethroid bifenthrin, motor impairment and loss of tyrosine hydroxylase‐positive neurons were paralleled by increased expression of transferrin receptor 2 (TfR2) in the substantia nigra (Zhang and Zhang 2024). Corroborating these experimental findings, Rhodes et al. (2014) provided compelling human genetic evidence by identifying protective haplotypes in the transferrin (TF) and transferrin receptor 2 (TFR2) genes against PD development, suggesting the TF‐TFR2 complex plays a key etiological role through iron misregulation. Considering the identification of transferrin receptor 1 (TfR1) as a selective surface marker for ferroptotic cells (Feng et al. 2020), the aforementioned evidence points to the transferrin receptor as a key player in iron overload‐mediated, ferroptosis‐induced dopaminergic cell death.

Concurrently, a compromised cellular capacity to export iron also seems to contribute to this pathological process. Ferroportin 1 (Fpn1), a primary cellular iron exporter, is downregulated in the PD brain (Raha et al. 2022). Further, in an experimental study, atrazine exposure in Wistar rats led to increased midbrain iron levels and dopaminergic neurotoxicity, events paralleled by decreased ferroportin expression (Li et al. 2021). This finding is particularly relevant given the established association between herbicide exposure and an increased risk of PD (James and Hall 2015).

The aforementioned evidence indicates that a combination of increased iron import and impaired export creates an appropriate environment for iron‐dependent ferroptosis, resulting in dopaminergic cell death. Notably, the consistent observation of elevated total iron in the SNpc, which correlates with disease severity (Dexter et al. 1987; Riederer et al. 1989), strongly suggests that a failure to maintain iron homeostasis is a core component of PD pathology. This iron dysregulation potentially drives dopaminergic neurodegeneration through ferroptosis.

#### 3.3.2 Lipid Peroxidation

A convergence of evidence from both experimental models and human studies confirms that lipid peroxidation is a critical pathological event in PD. In experimental PD models, dopaminergic neurodegeneration is consistently associated with elevated lipid peroxidation (Lin et al. 2022; Parkhe et al. 2020; Wang et al. 2025; Yao et al. 2024). A central regulator of this process is the enzyme glutathione peroxidase 4 (GPx4), which reduces toxic lipid hydroperoxides to inert lipid alcohols using GSH as an essential cofactor (Friedmann Angeli et al. 2014; Yang et al. 2014). Impaired GPx4 activity is a recurrent finding in these models. For instance, in both in vivo and in vitro 6‐hydroxydopamine (6‐OHDA) models, dopaminergic cell death coincides with a marked downregulation of GPx4 and increased lipid peroxidation (Huang et al. 2022; Sun et al. 2020). This mechanistic link is further reinforced by interventions that stabilize the GPx4 pathway; for example, activation of the AKT–GSK3β–GPx4 axis protects SH‐SY5Y cells from ferroptotic death in an MPTP model (Dong et al. 2025). Notably, in addition to GPx4, its essential cofactor GSH, which provides the electrons for reducing lipid hydroperoxides, is also decreased in a myriad of experimental PD models, where its loss is consistently associated with increased lipid peroxidation (Chao et al. 2018; Parkhe et al. 2020; Vora et al. 2022).

The clinical relevance of lipid peroxidation in human PD is also established. Foundational postmortem work by Dexter et al. (1986) provided the first direct evidence in human tissue, indicating that dopaminergic neuronal death in the substantia nigra is linked to excessive lipid peroxidation, as measured by malondialdehyde levels. This landmark finding positioned lipid peroxidation as a key terminal event in the PD neurodegenerative cascade. Subsequent studies have corroborated this, showing significant increases in additional lipid peroxidation markers, such as the metabolite 4‐hydroxy‐2‐nonenal (4‐HNE), in the cerebrospinal fluid of PD patients (Selley 1998). Notably, the decreased GSH levels reported in the substantia nigra of PD patients likely contribute directly to the observed increased lipid peroxidation in this structure (Jenner et al. 1992).

Remarkably, increased lipid peroxidation was documented in the brains of rodents in PD models (McCormack et al. 2005; Oida et al. 2006) and in PD patients (Dexter et al. 1986; Jenner et al. 1992; Selley 1998) well before the conceptualization of ferroptosis. Together, these human data (Dexter et al. 1986; Jenner et al. 1992; Selley 1998) validate the pathological sequence observed in models (McCormack et al. 2005; Oida et al. 2006) and underscore lipid peroxidation as a compelling therapeutic target. Thus, while strategies to counteract lipid peroxidation were already considered promising for mitigating dopaminergic neurodegeneration in the past, our current understanding of ferroptosis and its regulatory molecules makes this therapeutic possibility substantially more concrete and likely to succeed.

#### 3.3.3 α‐Synuclein

Alpha‐synuclein (α‐syn) is a presynaptic protein whose normal physiological functions, while not fully elucidated, are believed to include the regulation of synaptic vesicle trafficking and neurotransmitter release (Sharma and Burré 2023). In PD, however, α‐syn aggregates into toxic oligomers that disrupt cellular homeostasis, leading to neuronal death (Stefanis 2012). Notably, aggregated α‐syn and iron are both found to be present in Lewy bodies (Peng et al. 2010), a pathological hallmark of PD. The pathogenic process mediated by α‐syn can propagate through neural circuits, as secreted α‐syn can template the aggregation of native protein in recipient cells (Stefanis 2012).

A robust and pathological bidirectional relationship exists between α‐syn and cellular iron metabolism, creating a pro‐ferroptotic environment. Iron (in both Fe^2+^ and Fe^3+^ states) potently accelerates α‐syn aggregation by inducing conformational changes in the protein (Peng et al. 2010; Uversky et al. 2001), while conversely, α‐syn itself can modulate neuronal iron homeostasis. The 5′UTR of α‐syn mRNA contains an iron response element (IRE), allowing for translational upregulation of α‐syn in response to high iron levels (Friedlich et al. 2007). Moreover, Baksi et al. (2016) proposed that α‐syn directly mediates iron metabolism by colocalizing with TfR1 to facilitate iron uptake. Indeed, α‐syn depletion disrupts this process, causing TfR1 retention in endosomes and a reduction in cellular iron (Baksi et al. 2016). Furthermore, α‐syn has been proposed to possess ferrireductase activity, converting Fe^3+^ to the more redox‐active Fe^2+^ (Davies et al. 2011). This iron dysregulation, in turn, fuels the Fenton reaction, generating reactive oxygen species that promote the lipid peroxidation central to ferroptosis execution. Critically, iron chelation has been shown to reduce α‐syn pathology and rescue behavioral deficits in animal models, highlighting the therapeutic potential of targeting this axis (Carboni et al. 2017; Finkelstein et al. 2016).

Beyond iron, α‐syn is closely linked to lipid metabolism, directly influencing the membrane composition that determines cellular susceptibility to ferroptosis. Specifically, α‐syn binds to fatty acids (Sharon et al. 2001) and forms soluble oligomers in the presence of polyunsaturated fatty acids (PUFAs) (Sharon, Bar‐Joseph, Frosch, et al. 2003), the primary substrates for lipid peroxidation. A follow‐up study systematically confirmed this relationship, demonstrating elevated PUFA levels both in the brains of patients with PD and dementia with Lewy bodies, and in mesencephalic neuronal cells overexpressing α‐syn (Sharon, Bar‐Joseph, Mirick, et al. 2003). These findings support a model in which α‐syn drives intracellular PUFA accumulation to promote ferroptosis. This critical link was directly demonstrated in human iPSC‐derived neurons, where α‐syn oligomers incorporate into membranes, induce lipid peroxidation, and trigger aberrant calcium influx and cell death; this process was prevented by inhibiting lipid peroxidation or reducing iron‐dependent radicals (Angelova et al. 2020). Furthermore, reducing α‐syn expression confers ferroptosis resistance by altering the neuronal lipidome, specifically by lowering the levels of ether‐linked phospholipids that are required for ferroptosis execution (Mahoney‐Sanchez et al. 2022).

In conclusion, a compelling and convergent body of evidence points to ferroptosis as a potential key executioner pathway in α‐synuclein‐mediated dopaminergic neurodegeneration. The cumulative evidence (Baksi et al. 2016; Davies et al. 2011; Peng et al. 2010; Sharon, Bar‐Joseph, Mirick, et al. 2003; Sharon, Bar‐Joseph, Frosch, et al. 2003; Uversky et al. 2001) supports the concept of a pathological vicious cycle between α‐syn, iron, and PUFAs, wherein α‐syn aggregation drives iron dysregulation and lipid peroxidation, thereby promoting further aggregation and neuronal death. This is supported by interventional studies showing that ferroptosis inhibitors like ferrostatin‐1, iron chelators, and liproxstatin‐1 can mitigate α‐syn aggregation, protect mitochondria, and prevent cell death across multiple models, including rotenone‐treated SH‐SY5Y cells, *Drosophila*, and a mouse model with the overexpression of human α‐synuclein (Agostini et al. 2023; Kabiraj et al. 2015; Zhang et al. 2024).

Based on the preceding sections (3.3.1, 3.3.3), a mechanistic basis for the selective vulnerability of dopaminergic neurons (particularly those of the SNpc) to ferroptosis can be proposed, centering on their high basal metabolic rate, iron dyshomeostasis, and α‐syn pathology. These neurons exhibit a sustained Ca^2+^‐dependent pacemaking activity that drives a chronically elevated mitochondrial reactive oxygen species (ROS) flux, establishing a persistent prooxidant environment (Guzman et al. 2010). While the primary mitochondrial ROS is superoxide, a relatively low‐reactive species, its rapid dismutation to hydrogen peroxide (H_2_O_2_) sets the stage for ferroptosis susceptibility. A key determinant is the availability of labile iron (discussed in Section 3.3.1), which, via the Fenton reaction, converts H_2_O_2_ to the highly reactive hydroxyl radical (˙OH). Although H_2_O_2_ can induce apoptosis (Pallepati and Averill‐Bates 2011), the unique vulnerability of SNpc neurons to ferroptosis is explained by their elevated iron content, which favors ˙OH production and subsequent lipid peroxidation. Unlike radicals with specific biomolecular targets (e.g., thiyl radicals preferentially react with proteins; Tweeddale et al. 2007), ˙OH has an extremely short half‐life and seems to react indiscriminately with lipids, proteins, and nucleic acids. However, on average, its oxidative impact is disproportionately greater on lipids due to the chain propagation reaction: a single hydroxyl radical can initiate the oxidation of numerous PUFA‐containing phospholipids (Sevanian and Ursini 2000), thereby amplifying oxidative damage to a degree not observed with protein or nucleic acid targets.

This pro‐ferroptotic milieu is further exacerbated by α‐syn, a protein highly enriched in these neurons (Butler et al. 2017). α‐syn promotes iron uptake via transferrin receptor 1 colocalization and exhibits ferrireductase activity, converting Fe^3+^ to the more redox‐active Fe^2+^, thereby fueling Fenton chemistry (Baksi et al. 2016; Davies et al. 2011). Concurrently, α‐syn binds fatty acids and drives the accumulation of PUFAs (the primary substrates for lipid peroxidation), further sensitizing neurons to ferroptosis (Sharon et al. 2001). Adding another layer of specificity, ALOX15, a lipoxygenase enzyme that boosts ferroptosis in neurons (Zhao et al. 2022), is upregulated by α‐syn (Lin et al. 2023), directly linking a key pathological protein to the enzymatic machinery that drives phospholipid hydroperoxide accumulation. Collectively, these interconnected features (metabolic demand, iron dysregulation, α‐syn‐mediated alterations in iron and lipid handling, and the engagement of pro‐ferroptotic enzymes) explain why oxidative stress in dopaminergic neurons preferentially culminates in phospholipid hydroperoxide accumulation rather than in predominant protein oxidation or the activation of canonical mitochondrial apoptotic pathways.

Notably, ROS such as superoxide anion can stimulate the release of iron from aconitase (Castro et al. 2019), a process that directly fuels ferroptosis by expanding the intracellular pool of redox‐active iron. Integrating this with the features outlined above, a pathogenic sequence underlying the selective vulnerability of dopaminergic neurons can be proposed: autonomous pacemaking drives sustained Ca^2+^ influx, which in turn elevates mitochondrial ROS production (e.g., superoxide anion). These ROS promote the expansion of the labile iron pool, partly through iron release from aconitase, thereby setting the stage for Fenton chemistry (superoxide‐derived H_2_O_2_ + iron) and lipoxygenase (ALOX15)‐mediated oxidation of PUFAs. The resulting phospholipid hydroperoxide accumulation ultimately culminates in ferroptotic membrane failure. This integrated framework positions ferroptosis not as a single insult, but as the terminal executioner of a cascade of interdependent vulnerabilities inherent to the dopaminergic neuron, and highlights the multiple nodes within this sequence that may be therapeutically targeted.

**FIGURE 3:**
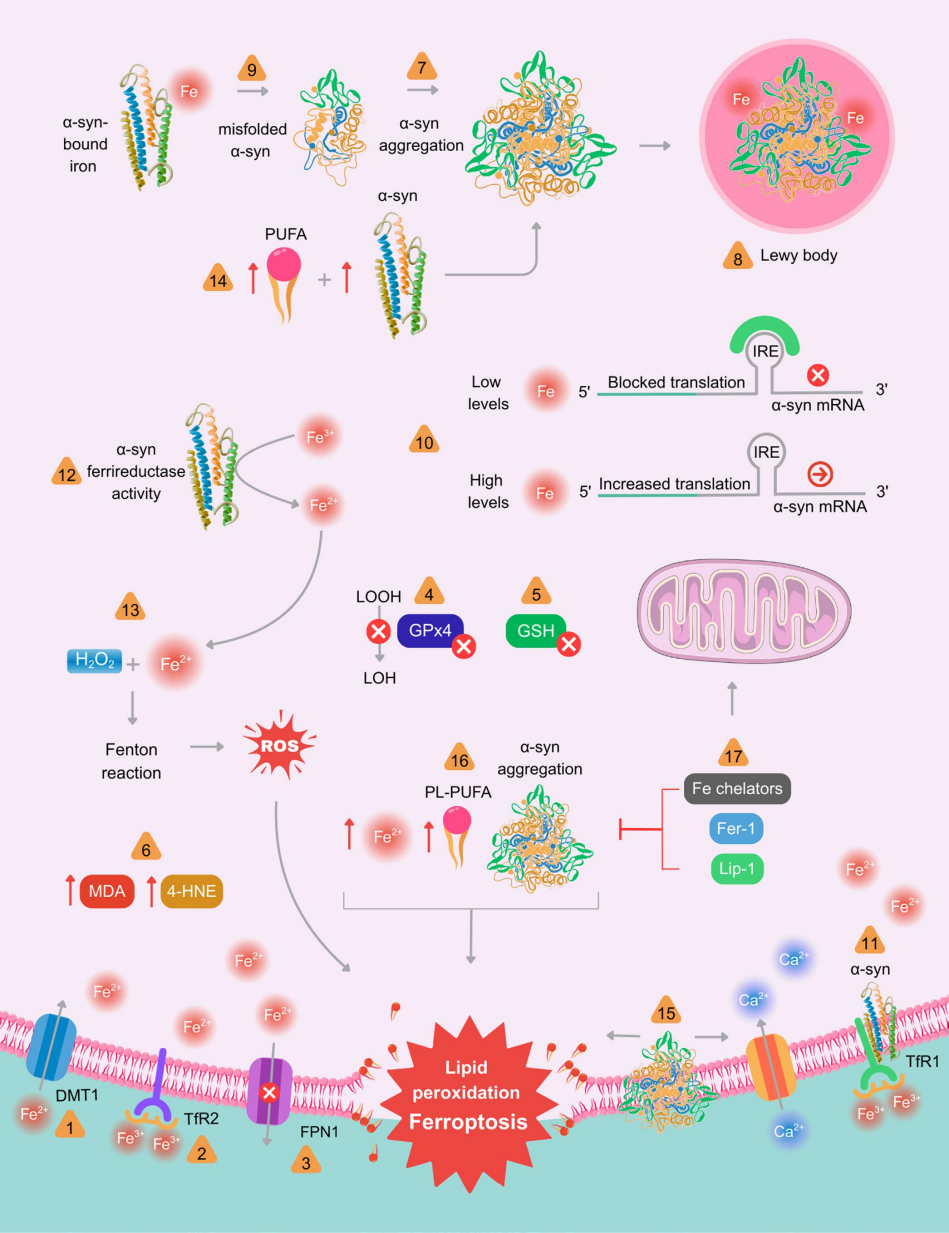
Events linking iron dysregulation, alpha‐synuclein, oxidative stress, and ferroptosis to dopaminergic neurodegeneration. Iron dyshomeostasis: Divalent metal transporter 1 (DMT1) expression is upregulated in the ventral mesencephalon of 1‐methyl‐4‐phenyl‐1,2,3,6‐tetrahydropyridine (MPTP)‐intoxicated mice and in the substantia nigra of Parkinson's disease (PD) patients (*event 1*). Likewise, transferrin receptor 2 (TfR2) expression increases in the substantia nigra after exposure to MPTP and the pyrethroid bifenthrin (*event 2*). Ferroportin 1 (FPN1) is downregulated in the PD brain. Wistar rats treated with atrazine produced midbrain iron (Fe) accumulation and dopaminergic neurotoxicity that paralleled decreased ferroportin expression (*event 3*). Lipid peroxidation: In 6‐hydroxydopamine (6‐OHDA) models (both in vitro and in vivo), dopaminergic cell death coincides with marked downregulation of GPx4 and increased lipid peroxidation (*event 4*). Glutathione (GSH) is depleted across many PD experimental models (*event 5*); this loss is consistently linked to elevated lipid peroxidation. Postmortem PD brain tissue shows increased malondialdehyde (MDA), and cerebrospinal fluid from PD patients contains elevated 4‐hydroxy‐2‐nonenal (4‐HNE) (*event 6*). α‐synuclein: In PD, α‐synuclein (α‐syn) aggregates into toxic oligomers that disrupt cellular homeostasis, leading to neuronal death (*event 7*). Aggregated α‐syn and iron are both found to be present in Lewy bodies, a pathological hallmark of PD (*event 8*). Iron (in both Fe^2+^ and Fe^3+^ states) potently triggers α‐syn aggregation by inducing conformational changes in the protein (*event 9*). The 5′UTR of α‐syn mRNA contains an iron response element (IRE), allowing for translational upregulation of α‐syn in response to high iron levels (*event 10*). α‐Syn can colocalize with transferrin receptor‐1 (TfR1) to facilitate iron uptake (*event 11*) and has been proposed to possess ferrireductase activity, potentially converting Fe^3+^ to the more redox‐active Fe^2+^ (*event 12*). Convergence: iron, lipids, and α‐synuclein form a pathogenic loop. Increased labile iron promotes the Fenton reaction (*event 13*), generating reactive oxygen species (ROS) that drive lipid peroxidation, the biochemical execution pathway of ferroptosis. α‐syn binds to fatty acids and forms soluble oligomers in the presence of polyunsaturated fatty acids (PUFAs) (*event 14*). α‐syn oligomers incorporate into membranes, induce lipid peroxidation, and trigger aberrant calcium (Ca^2+^) influx and cell death (*event 15*). The processes create a pathological vicious cycle between α‐syn, iron, and PUFAs, wherein α‐syn aggregation drives iron dysregulation and lipid peroxidation, thereby promoting further aggregation and neuronal death (*event 16*). Compounds such as ferrostatin‐1 (Fer‐1), iron chelators, and liproxstatin‐1 (Lip‐1) can mitigate α‐syn aggregation, protect mitochondria, and prevent cell death across multiple models (*event 17*). Events 1–17 are indicated as orange triangles. References for the aforementioned events are present in the main text. Fe^2+^, ferrous iron; Fe^3+^, ferric iron; H_2_O_2_, hydrogen peroxide; LOH, lipid alcohols; LOOH, lipid hydroperoxides; PL‐PUFA, phospholipids containing polyunsaturated fatty acyl tails.

Figure 3 depicts the major events linking iron dysregulation, lipid peroxidation, α‐synuclein, and oxidative stress in ferroptosis‐mediated dopaminergic neurodegeneration.

#### 3.3.4 Ferroptosis Beyond PD: NBIA Disorders

In addition to PD, the potential role of ferroptosis in neurodegeneration is illustrated by a group of inherited disorders known as Neurodegeneration with Brain Iron Accumulation (NBIA), which represent a heterogeneous group of disorders characterized by pathological iron deposition, primarily in the globus pallidus and substantia nigra, leading to a clinical presentation that often includes parkinsonism, spasticity, and dystonia (Wong and Krainc 2017). These conditions provide compelling human genetic evidence that primary disruptions in iron metabolism are sufficient to drive progressive neurological decline, offering unique insights into the core mechanisms of ferroptosis. Notably, cells other than dopaminergic neurons are also involved in NBIA pathology.

A key example of NBIA is pantothenate kinase‐associated neurodegeneration (PKAN), an autosomal‐recessive disorder caused by mutations in the *PANK2* gene. This gene encodes the mitochondrial enzyme pantothenate kinase‐2, which catalyzes the crucial first step in coenzyme A (CoA) biosynthesis. The resulting impairment disrupts cellular metabolism and leads to the hallmark of the disease: pathological iron accumulation within specific brain cells. Research using iPSC‐derived models has been instrumental in elucidating this process. Notably, while iron deposition occurs in both GABAergic neurons and glial cells, GABAergic neurons suffer significantly reduced viability (Santambrogio et al. 2022). Furthermore, PKAN astrocytes demonstrate a particularly pronounced iron overload, with approximately 50% of cells affected, alongside severe alterations in mitochondrial morphology, respiratory function, and oxidative status. Critically, these PKAN astrocytes exhibit clear molecular signs of ferroptosis and adopt a neurotoxic phenotype, which was directly demonstrated in coculture experiments where they compromised the survival of glutamatergic neurons. This evidence strongly suggests that the convergence of impaired CoA‐dependent lipid metabolism and iron accumulation creates an ideal substrate for ferroptosis, driving neurodegeneration in PKAN (Santambrogio et al. 2022).

Another highly informative NBIA disorder is neuroferritinopathy, which results from mutations in the *FTL* gene encoding ferritin light chain and is characterized by iron and ferritin aggregate accumulation in the brain. This disorder directly disrupts the cell's primary iron storage capacity, underscoring the essential role of ferritin in maintaining brain iron homeostasis (McNally et al. 2019). A failure of central nervous system iron buffering is also evidenced by markedly reduced cerebrospinal fluid ferritin levels, which occur despite typically normal serum ferritin (Nishida et al. 2014). This loss of iron sequestration inevitably leads to an increase in the redox‐active iron pool, predisposing neurons to iron‐catalyzed oxidative damage. Crucially, studies using patient‐derived neurons have demonstrated that this iron dysregulation is sufficient to cause both cellular senescence and ferroptotic cell death, providing direct evidence that non‐ferritin‐bound iron is a primary driver of neuronal degeneration (Cozzi et al. 2019). The recent identification of pediatric cases with de novo *FTH1* mutations, presenting with developmental delay, epilepsy, and iron accumulation, further highlights the critical nature of ferritin function in neuroprotection (Shieh et al. 2023). The genetic landscape of NBIA extends to other key pathways. Mutations in PLA2G6 (encoding a calcium‐independent phospholipase A2β; iPLA2β) and C19orf12 (encoding a mitochondrial membrane protein) are also linked to NBIA and are often associated with the presence of Lewy bodies, directly connecting these forms of iron dyshomeostasis to synuclein pathology (Arber et al. 2016). In line with this, a recent in vivo experimental study demonstrated that iPLA2β deficiency in dopaminergic neurons induces ferroptosis, aggravating neurodegeneration and motor deficits in a mouse model of PLA2G6‐associated neurodegeneration (Li, Liu, et al. 2025). Moreover, a parallel mechanistic study on Mitochondrial Membrane Protein‐Associated Neurodegeneration (MPAN), caused by *C19orf12* mutations, has revealed a similar pathogenic cascade. Research using patient‐derived fibroblasts and neuronal models demonstrated that *C19orf12* deficiency leads to mitochondrial fragmentation, iron overload, and oxidative damage, rendering cells highly susceptible to ferroptosis. Crucially, this ferroptotic cell death was preventable by the iron chelator deferoxamine, directly implicating iron‐driven ferroptosis as a central mechanism in MPAN pathogenesis (Shao et al. 2022).

Collectively, the study of NBIA disorders provides fundamental validation of the ferroptosis pathway in vivo. They demonstrate that genetic lesions affecting diverse processes, from iron storage (ferritinopathies) to lipid metabolism (PKAN, PLA2G6‐associated neurodegeneration) and organelle function, can converge to promote pathological iron accumulation and create a cellular environment exquisitely vulnerable to ferroptotic cell death. It is important to note, however, that the pathogenic drivers of ferroptosis in NBIA disorders may differ from those central to PD and related synucleinopathies. In PD, ferroptosis is propelled by a multihit vulnerability that affects mostly dopaminergic neurons of the substantia nigra pars compacta (SNpc), where high iron content, dopamine metabolism, and α‐synuclein pathology synergize to create a uniquely prooxidant milieu. In contrast, NBIA disorders are primarily initiated by germline mutations causing systemic iron‐metabolism defects, leading to widespread iron overload that affects multiple neural cell types across broader brain regions. While parkinsonism can be a clinical feature of NBIA, the pathology in disorders like PKAN is not fundamentally linked to α‐synuclein aggregation (Li et al. 2013), highlighting a distinct etiological pathway that culminates in the shared endpoint of iron‐dependent neurodegeneration.

## 4 Potential Therapeutic Strategies and Translational Challenges

### 4.1 Preclinical Models Supporting Ferroptosis Inhibition as a Neuroprotective Strategy

Since the initial discovery and conceptualization of ferroptosis and the key reagents to study it (Dixon et al. 2012), and its subsequent implication in the pathogenesis of neurodegenerative diseases such as Parkinson's disease and related conditions (Mahoney‐Sánchez et al. 2021; Stockwell et al. 2017), there has been a substantial and rapid expansion of experimental and preclinical research on this research topic. A significant body of this work demonstrates that pharmacological inhibition of ferroptosis, achieved not only with canonical inhibitors like ferrostatin‐1 and liproxstatin‐1 but also with a diverse array of compounds possessing secondary anti‐ferroptotic activity, confers protection against dopaminergic neuronal loss (detailed in the next paragraphs of this subsection). Furthermore, the protective effects of established ferroptosis inhibitors have been consistently replicated across classical Parkinson's disease models utilizing neurotoxins such as 1‐methyl‐4‐phenyl‐1,2,3,6‐tetrahydropyridine (MPTP), 1‐methyl‐4‐phenylpyridinium (MPP^+^), rotenone and 6‐hydroyxdopamine (6‐OHDA) (Bai et al. 2021; Huang et al. 2022; Kabiraj et al. 2015; Li, Zhang, et al. 2023; Sun et al. 2020). Furthermore, a growing number of compounds not originally designed to target ferroptosis (e.g., chelating agents, antioxidants, and anti‐inflammatory agents) have been shown to exert their protective effects against dopaminergic cell death, at least in part, through the inhibition of ferroptotic pathways in both in vitro and in vivo models. These are discussed in the next paragraphs.

In rodent models of dopaminergic neurodegeneration utilizing neurotoxins like MPTP, 6‐OHDA, and rotenone, a diverse range of compounds has demonstrated protective effects at least partly due to anti‐ferroptotic properties. These include natural compounds like alpha‐lipoic acid (Wang et al. 2025), muscone (Xu et al. 2025), anthocyanins from blue honeysuckle (Li, Fan, et al. 2025), and myricetin (Gu et al. 2024), as well as clinically relevant molecules like the dipeptidyl peptidase‐4 (DPP‐4) inhibitor teneligliptin (Huang et al. 2025) and the antibiotic ceftriaxone (Zhi et al. 2025). The protective effects are consistently evidenced by the amelioration of motor deficits, preservation of dopaminergic neurons in the substantia nigra, and reduction of key pathological markers like α‐synuclein aggregation.

Complementary in vitro models using MPP^+^, 6‐OHDA, or erastin in dopaminergic cell lines such as SH‐SY5Y, PC12, and LUHMES cells confirm the direct cytoprotective effects of these compounds and other nonclassical ferroptosis inhibitors against ferroptosis (Chen et al. 2025; Gutbier et al. 2020; Güzel et al. 2025; Kong et al. 2024; Li, Fan, et al. 2025; Sun et al. 2025). Furthermore, in vivo studies in nonmammalian models have been instrumental in demonstrating the evolutionary conservation of this pathway. For example, epigallocatechin‐3‐gallate (EGCG) was shown to mitigate PD phenotypes in PINK1 mutant flies by restoring iron homeostasis (Xia et al. 2024). Parallel work in *Caenorhabditis elegans* confirmed that ferrostatin‐1 rescues dopaminergic neurons from iron‐induced, ferroptosis‐like death (Ferreyra et al. 2024). These studies validate nonmammalian models as useful tools for probing ferroptosis mechanisms and provide a basis for more complex future investigations.

The mechanistic underpinnings of the protection afforded by anti‐ferroptotic strategies against dopaminergic cell damage are multifaceted, primarily converging on the enhancement of cellular antioxidant defenses and the restoration of iron homeostasis. Activation of the NRF2 (nuclear factor erythroid 2‐related factor 2) pathway emerges as a central and highly targeted mechanism. Numerous compounds, including alpha‐lipoic acid, cyanidin‐3‐O‐glucoside, salidroside, morroniside, and quercetin, exert their effects by promoting NRF2 nuclear translocation, thereby upregulating a suite of cytoprotective genes (Li, Zhang, et al. 2023; Jiang et al. 2023; Li, Fan, et al. 2025; Shen et al. 2024; Wang et al. 2025). This includes heme oxygenase‐1 (HMOX1), glutathione peroxidase 4 (GPx4), the cystine/glutamate antiporter (xCT/SLC7A11), and ferritin heavy chain 1 (FTH1), collectively reducing oxidative stress and inhibiting lipid peroxidation. A second critical mechanism involves the regulation of iron metabolism to prevent the deleterious accumulation of labile iron. Compounds such as buddlejasaponin Ivb (Li, Xu, et al. 2024) and the PPARδ agonist GW501516 (Lee et al. 2022) achieve this by modulating the expression of iron regulatory proteins (IRPs) and transporters like Divalent Metal Transporter 1 (DMT1) and ferroportin. A sophisticated nanomedicine approach utilizing engineered deferoxamine nanosheets (BDPR NSs) was designed to specifically sequester iron within the labile iron pool, demonstrating significant neuroprotection under in vivo conditions (Lei et al. 2024). Furthermore, a strategy involves the inhibition of ferritinophagy, the autophagic degradation of ferritin that releases stored iron. Clozapine‐N‐oxide, ganoderic acid A, and JWA protein were all shown to protect neurons by suppressing NCOA4‐mediated ferritinophagy, thus preventing iron overload and subsequent ferroptosis (Shen et al. 2024; Sun et al. 2024; Zhao et al. 2024).

Beyond these core pathways, several other targeted mechanisms have been identified. Direct inhibition of lipid peroxidation is achieved by targeting enzymes like the lipoxygenase ALOX5; the compound (−)‐clausenamide was found to bind directly to ALOX5, preventing its nuclear translocation and the production of pro‐ferroptotic lipid metabolites (Li, Wang, et al. 2023). The induction of mitophagy works in concert with these pathways; acteoside and a neutrophil‐inspired nanozyme were reported to enhance the clearance of damaged mitochondria, thereby reducing a major source of reactive oxygen species and mitigating ferroptosis (Han et al. 2024; Tian et al. 2025). Moreover, several studies highlight the role of nonclassical ferroptosis inhibitors. Notably, the hydroxyindole class of compounds, including 3‐hydroxyindole, were identified as potent ferroptosis inhibitors acting via intrinsic radical‐trapping antioxidant activity in cultured N27 (rat dopaminergic neurons) cell line (Jakaria and Cannon 2025).

In conclusion, converging preclinical evidence establishes ferroptosis as a central mechanism of dopaminergic neuronal death, with the protective effects of diverse compounds consistently converging on the same core endpoints: reduction of the labile iron pool, attenuation of PUFA‐phospholipid peroxidation, and preservation of the GSH‐GPx4 antioxidant axis. Whether through direct radical trapping (e.g., ferrostatin‐1, liproxstatin‐1), or activation of the NRF2 transcriptional program to bolster GSH synthesis and iron sequestration, these interventions demonstrate that pharmacological interruption of the ferroptotic cascade is sufficient to confer neuroprotection. This strong mechanistic rationale, rooted in the specific molecular hallmarks of ferroptosis, is now driving translational efforts to determine whether these preclinical successes can be recapitulated in patients.

### 4.2 Overview of Clinical Trial Data

Here we report on completed or ongoing clinical trials investigating therapies targeting ferroptosis and/or iron dysregulation for PD and related neurodegenerative disorders characterized by dopaminergic cell loss.

The low‐molecular‐weight antioxidant peptide GSH is a pivotal inhibitor of ferroptosis. This role is attributed not only to its function as an essential cofactor for GPx4, a key ferroptosis regulator (Friedmann Angeli et al. 2014; Yang et al. 2014), but also to its ability to directly chelate cytosolic Fe(II) (Bayır et al. 2020; Hider and Kong 2011), thereby mitigating iron‐driven oxidative damage. Given this central role, strategies to elevate brain GSH levels, including the administration of its precursors N‐acetylcysteine (NAC) and gamma‐glutamylcysteine (GGC), have been investigated in clinical trials for PD. In one study (NCT01427517, https://www.clinicaltrials.gov/study/NCT01427517), intravenous NAC increased blood GSH redox ratios and, subsequently, brain GSH levels in participants with PD and Gaucher disease (Holmay et al. 2013). However, a separate trial of oral NAC (NCT02212678, https://www.clinicaltrials.gov/study/NCT02212678) reported a disconnect between peripheral and central effects: while peripheral antioxidant markers improved, neither brain GSH levels nor markers of lipid peroxidation (4‐HNE and MDA) were significantly altered (Coles et al. 2018). Consequently, while these studies demonstrate the feasibility of modulating peripheral GSH metabolism, they do not establish a beneficial effect of NAC on dopaminergic neurodegeneration or clinical outcomes in PD.

According to a recent clinical trial listing (NCT07064005, https://www.clinicaltrials.gov/study/NCT07064005), the efficacy of oral GGC supplementation is under investigation for its potential to enhance brain glutathione levels, reduce iron accumulation, and mitigate ferroptotic stress. In another approach, intranasal GSH administration failed to demonstrate superiority over placebo in a 3‐month clinical trial (NCT02424708, https://www.clinicaltrials.gov/study/NCT02424708), despite showing some predicted improvements in PD motor and total scores. These clinical trials (NCT01427517, NCT02212678, NCT07064005, NCT02424708) indicate that boosting GSH is a plausible strategy for PD; however, its efficacy has not been demonstrated, and the biological pathway by which it might exert its effect, including any link to ferroptosis, remains unknown.

As already discussed, ubiquinone (coenzyme Q_10_, CoQ_10_) and vitamin E (α‐tocopherol) are lipid‐soluble antioxidants that can inhibit ferroptosis through their radical‐trapping activity. In its reduced form (ubiquinol), CoQ_10_ acts as a potent trap for lipid peroxyl radicals, thereby halting the propagation of lipid peroxidation (Doll et al. 2019). Similarly, vitamin E and related compounds, such as tocotrienols, function primarily as peroxyl radical scavengers to suppress ferroptotic cell death (Hinman et al. 2018; Hu et al. 2021; Saito 2021). This common mechanistic rationale has prompted the clinical evaluation of these compounds in PD. A phase III clinical trial (NCT00740714, https://clinicaltrials.gov/study/NCT00740714) investigated high‐dose CoQ_10_ (up to 2400 mg/day) in PD patients. While the treatment was safe, it failed to demonstrate clinical benefit (The Parkinson Study Group QE3 Investigators et al. 2014), despite promising results from an earlier phase II study that suggested a slowing of disease progression (Shults 2002). For vitamin E derivatives, the impetus for clinical translation comes not only from their radical‐trapping mechanism but also from supportive preclinical data; indeed, studies on tocotrienols have demonstrated neuroprotective effects in models of PD (Matsura 2019; Nakaso et al. 2016). This evidence underpins an ongoing randomized clinical trial (NCT04491383, https://clinicaltrials.gov/study/NCT04491383) assessing the effects of tocotrienols on motor and non‐motor symptoms in PD patients; the trial is currently recruiting participants. In summary, despite the strong biological rationale for using CoQ_10_ and vitamin E to target ferroptosis in PD, clinical evidence of their efficacy is lacking, and a connection to ferroptosis inhibition in patients remains unproven, warranting further study.

Iron chelation represents a direct strategy to inhibit ferroptosis by reducing lipid peroxidation (Dixon et al. 2012). The neuroprotective and neurorestorative potential of this approach has been established in preclinical PD models using brain‐permeable chelators (Bar‐Am et al. 2015; Zeng et al. 2021; Zhu et al. 2007). Translating this rationale to the clinic, the brain‐penetrant iron chelator deferiprone has been evaluated in several clinical trials. Initial pilot studies (NCT01539837, https://clinicaltrials.gov/study/NCT01539837; NCT00943748, https://clinicaltrials.gov/study/NCT00943748) demonstrated that deferiprone effectively reduces cerebral iron accumulation in regions such as the substantia nigra, with some reports of associated clinical improvement. However, subsequent larger, randomized controlled trials have yielded conflicting outcomes. The FAIRPARK‐II trial (NCT02655315, https://clinicaltrials.gov/study/NCT02655315) found that, despite successfully reducing nigrostriatal iron levels, deferiprone treatment worsened parkinsonian symptoms compared to placebo (Devos et al. 2022). Another major trial (NCT02728843, https://clinicaltrials.gov/study/NCT02728843) concluded that deferiprone provided no significant benefit on its primary motor outcome (Devos et al. 2025). In summary, the clinical efficacy of iron chelation in PD appears dissociated from its ability to reduce brain iron. The discordant outcomes of deferiprone trials (specifically, effective reduction of nigral iron without clear clinical benefit or even with symptom worsening) constitute a critical paradox that warrants mechanistic explanation. Some non‐mutually exclusive hypotheses may account for this. One possibility is that the therapeutic window for iron chelation is inherently narrow. Deferiprone, a lipophilic chelator, may deplete not only the pathological labile iron pool but also essential iron pools required for the biosynthesis of iron–sulfur clusters and heme, as well as for the activity of tyrosine hydroxylase, the rate‐limiting enzyme in dopamine synthesis. Such off‐target depletion could inadvertently impair residual dopaminergic function, offsetting any potential benefit from reduced oxidative stress. In this regard, Devos et al. (2022) speculated that the early separation of disease progression curves favoring the placebo group might reflect a negative symptomatic effect of deferiprone rather than an acceleration of underlying neurodegeneration, a possibility supported by the observed increase in plasma prolactin, a marker consistent with reduced dopaminergic tone (Fitzgerald and Dinan 2008). A second hypothesis invokes a temporal limitation: by the time of clinical diagnosis, the ferroptotic cascade may have already crossed a point of no return, with substantial neuronal loss already sustained. In this advanced stage, iron reduction might halt further degeneration but cannot restore lost neurons, and any potential benefit may be overshadowed by the essential role of iron in supporting the function of surviving (although compromised) dopaminergic neurons. Dissecting these mechanistic possibilities is essential for the rational design of next‐generation therapies aimed at safely and effectively targeting ferroptosis without disrupting the fundamental iron‐dependent physiology of the brain.

Beyond PD, we extended our search for clinical trials targeting ferroptosis or iron dysregulation in other disorders involving dopaminergic cell loss, such as dementia with Lewy bodies and Neurodegeneration with Brain Iron Accumulation (NBIA). While no such trials were identified for dementia with Lewy bodies, the iron chelator deferiprone is under investigation for NBIA, including the subtype pantothenate kinase‐associated neurodegeneration (PKAN). Although recent trials (NCT00907283, https://clinicaltrials.gov/study/NCT00907283; NCT02635841, https://clinicaltrials.gov/study/NCT02635841) are still ongoing without posted results, findings from a completed study (NCT01741532, https://www.clinicaltrials.gov/study/NCT01741532) have demonstrated a significant effect. This trial provided the first clinical evidence that deferiprone can slow disease progression in patients with NBIA, specifically those with atypical PKAN (Klopstock et al. 2019). Despite the nascent state of clinical research into iron chelation for non‐PD dopaminergic neurodegeneration, the promising findings of the NCT01741532 trial affirm its potential as a viable treatment for disorders of iron dyshomeostasis. Nevertheless, the specific involvement of ferroptosis inhibition in mediating these clinical benefits remains to be conclusively established, highlighting an important direction for future research.

## 5 Major Translational Challenges, Future Directions, and Concluding Remarks

A compelling body of preclinical evidence firmly establishes ferroptosis as a central mechanism of dopaminergic neuronal death, and its pharmacological inhibition confers significant neuroprotection in models of PD and related conditions (see Section 4.1). In contrast, clinical trials translating these findings into therapies for PD and related disorders have largely yielded neutral or, in the case of some iron chelators, even negative outcomes (see Section 4.2). This translational disconnect underscores critical challenges and opportunities that we will explore in the following sections.

### 5.1 Challenges in Translating Experimental Models

The limited translatability of most experimental models remains a key challenge. Commonly used paradigms involve coadministering a neuroprotective agent (e.g., antioxidants, dopaminergic agonists) with an acute toxin (e.g., 6‐OHDA, MPTP, paraquat) to induce rapid neuronal death. This approach poorly mirrors the slow, complex pathogenesis of human PD, as well as related disorders such as NBIA and dementia with Lewy bodies. While valuable for high‐throughput screening, such paradigms fail to recapitulate the chronic progression of these diseases. Consequently, the observed “neuroprotection” may represent a direct pharmacological antagonism of the acute toxin rather than a genuine disease‐modifying effect. Although more complex and costly, preclinical models that better mimic the chronic progression of human disease (e.g., in vivo approaches featuring progressive neurodegeneration and symptom appearance) are needed to provide a more reliable foundation for clinical trials.

A further critical challenge is the timing of therapeutic intervention. PD is typically diagnosed only after substantial neurodegeneration has occurred; by the onset of symptoms, approximately 80% of putamenal dopamine is depleted and 60% of dopaminergic neurons in the substantia nigra pars compacta are lost (Dauer and Przedborski 2003). Therefore, therapeutic strategies must be evaluated for their ability to halt or even reverse established damage, an intimidating challenge for most interventions, including anti‐ferroptotic drugs. This timing issue likely limits the efficacy of even well‐founded treatments. While the rationale for targeting ferroptosis is strong, supported by evidence of cerebral iron dyshomeostasis, depleted glutathione, and elevated lipid peroxidation (Section 4.1), initiating treatment at this advanced stage may offer limited functional benefit. By the time of clinical diagnosis, the disease is likely sustained by a self‐perpetuating cascade of oxidative stress, impaired proteostasis, neuroinflammation, and mitochondrial dysfunction (Dong‐Chen et al. 2023), which may not be fully arrested by targeting a single pathway like ferroptosis. This complex pathophysiology likely explains, at least in part, the limited efficacy observed in clinical trials to date.

### 5.2 The Imperative of Drug‐Like Properties

The successful translation of neuroprotective compounds from experimental models to clinical practice is also critically dependent on favorable drug‐like properties, particularly the ability to cross the BBB and achieve adequate pharmacokinetics in humans. This remains a significant impediment for most first‐generation ferroptosis inhibitors.

The prototype compound ferrostatin‐1 (Fer‐1), a radical‐trapping agent, has been instrumental in elucidating ferroptosis mechanisms and demonstrating therapeutic potential in diverse disease models (Gaschler et al. 2018; Martín‐Saiz et al. 2022; Scarpellini et al. 2023). However, its utility as a lead molecule is limited by poor drug‐like properties, primarily a labile ester moiety that undergoes rapid hydrolysis to an inactive metabolite (Devisscher et al. 2018). Promisingly, novel approaches are emerging to overcome these limitations, including the development of water‐soluble Fer‐1‐based poly(2‐oxazoline)‐drug conjugates (Morrow et al. 2022) and the ongoing design of novel Fer‐1 derivatives with optimized pharmacological profiles (Wang et al. 2024).

Liproxstatin‐1, a spiroquinoxalinamine derivative with radical‐trapping properties, potently inhibits ferroptosis. It has shown efficacy in models beyond its initial discovery in acute renal failure (Friedmann Angeli et al. 2014), including subarachnoid hemorrhage (Cao et al. 2021), which suggests a potential for central action. Despite favorable pharmacokinetics in mice, its clinical applicability is contingent upon demonstrating effective blood–brain barrier penetration in humans, which requires further investigation.

The critical challenge of BBB penetration is further underscored by UAMC‐3203, a Fer‐1 analog engineered for better solubility and stability (Devisscher et al. 2018). This compound showed significantly stronger protection than Fer‐1 in a peripheral disease model (acute liver dysfunction) and achieved extensive tissue distribution in mice and rats. However, it was hardly detectable in the spinal fluid and brain, demonstrating minimal BBB crossing (Van Coillie et al. 2022). This case clearly illustrates that improving general pharmacokinetics is insufficient for neurodegenerative applications, underscoring the need for intentional molecular design to achieve central nervous system exposure.

### 5.3 Future Directions and Concluding Remarks

Despite the challenges of initiating anti‐ferroptotic therapy at advanced disease stages, where significant neurodegeneration may limit functional benefit, a broader analysis reveals two potential strategic utilities for this approach.

#### 5.3.1 Prophylactic or Early Intervention in Genetic Syndromes

For genetic forms of Parkinson's disease (PD) or Neurodegeneration with Brain Iron Accumulation (NBIA) disorders, where early diagnosis via genetic screening is feasible and dopaminergic neurodegeneration is a predictable outcome, anti‐ferroptotic agents could be deployed prophylactically or at the earliest symptomatic stages. In this context, strategies that bolster global antioxidant capacity (e.g., GSH precursors) or trap peroxyl radicals (e.g., liproxstatin‐1) may hold an advantage over iron chelation. These approaches could mitigate lipid peroxidation directly without disrupting the iron homeostasis essential for fundamental cellular processes.

#### 5.3.2 Adjunctive Therapy With Standard Treatments

A second strategy involves coadministering anti‐ferroptotic agents with standard dopaminergic treatments such as L‐DOPA. Evidence suggests that chronic L‐DOPA usage is associated with elevated nigral iron levels (Du et al. 2022). A combination therapy could therefore serve a dual purpose: managing motor symptoms while simultaneously counteracting a treatment‐induced increase in ferroptotic vulnerability. This approach has the potential to modify the disease course by protecting neurons from progressive damage.

In summary, while ferroptosis has emerged as a compelling pathogenic mechanism and source of druggable targets in dopaminergic neurodegeneration, its clinical translation faces significant hurdles. To succeed, the field must focus on developing human‐relevant models, compounds with optimized drug‐like properties, and precision clinical trials. The ultimate goal, and remaining paramount challenge, is to determine whether ferroptosis inhibition can actually alter the progressive course of PD and related conditions, such as NBIAs and dementia with Lewy bodies.

## Author Contributions

**Carmem L. Sperlich:** conceptualization, methodology, writing – original draft, writing – review and editing. **Brent R. Stockwell:** conceptualization, methodology, writing – original draft, writing – review and editing. **Marcelo Farina:** conceptualization, methodology, writing – original draft, writing – review and editing.

## Funding

This work was supported by National Institutes of Health (grant no. P30CA008748), Conselho Nacional de Desenvolvimento Científico e Tecnológico (CNPq‐Brazil grant 303121/2022‐0, 445770/2025‐2, 406442/2022‐3), Fundação de Amparo à Pesquisa e Inovação do Estado de Santa Catarina (FAPESC/TO/2024TR002498), Coordenação de Aperfeiçoamento de Pessoal de Nível Superior (Finance Code 001 and PDSE 88881.982640/2024‐01), National Institutes of Health (grant no. P30CA013696), National Institutes of Health (NIH grant 5P30DK132710), Office of the Director, National Institutes of Health (S10OD020056), and National Institutes of Health (grant nos. S10OD012351, S10OD021764, and S10OD032433).

## Conflicts of Interest

B.R.S. is an inventor on patents and patent applications involving ferroptosis, holds equity in and serves as a consultant to ProJenX Inc., and serves as a consultant to Weatherwax Biotechnologies Corporation. The other authors declare no conflicts of interest.

## Data Availability

Data sharing is not applicable to this article because no new data were created or analyzed in this study.
